# Maintenance of “stem cell” features of cartilage cell sub-populations during in vitro propagation

**DOI:** 10.1186/1479-5876-11-27

**Published:** 2013-01-30

**Authors:** Karin Benz, Claudia Stippich, Christian Freudigmann, Juergen A Mollenhauer, Wilhelm K Aicher

**Affiliations:** 1 NMI Natural and Medical Sciences Institute at the University of Tuebingen, Markwiesenstr. 55, Reutlingen, 72770, Germany; 2 TETEC AG, Reutlingen, Germany; 3 Rush University Medical Center, Chicago, IL, USA; 4 KFO273, Department of Urology, University of Tuebingen Hospital, Tuebingen, Germany

**Keywords:** Stem cells, Chondrocytes, Disc cells, Autologous chondrocyte implantation, Magnetic sorting, Differentiation

## Abstract

**Background:**

The discovery of mesenchymal stem cells (MSCs) or MSC-like cells in cartilage tissue does not tie in well with the established view that MSCs derive from a perivascular niche. The presence of MSCs may raise concerns about specificity and application safety, particularly in terms of the regulatory site. The aim of the present study was to investigate the benefits or possible risks of the MSC-like properties of cells isolated from cartilage in the context of autologous chondrocyte implantation.

**Methods:**

Chondrocytic cells were isolated from cartilage or intervertebral disc tissue. Flow cytometry was used to analyze the expression of cell surface antigens. MSC-like cells were either enriched or depleted by means of magnetic cell sorting (MACS) involving the monoclonal antibodies W5C5/SUSD2 and W8B2/MSCA-1. We addressed the issues of prolonged expansion of such cells as well as the influence of culture medium as a trigger for selecting a single cell type. Established protocols were used to study *in vitro* differentiation. In addition to histological and biochemical assessment, the acquired phenotypes were also evaluated on the mRNA transcript level.

**Results:**

In the studied cells, we found strongly analogous expression of antigens typically expressed on MSCs, including CD49e, CD73, CD90, CD105, CD140b and CD166. The expression of W5C5 and W8B2 antigens in cartilage cell sub-populations did not correlate with multi-potency. We demonstrated that a chondroid precursor, but not a bona fide multipotent mesenchymal, cell type can be obtained under established *in vitro* culture conditions. The culture media used for expansion influenced the cell phenotype.

**Conclusions:**

The risk of adverse adipose or osseous differentiation is not posed by expanded chondrocyte cultures, even after enrichment of putative MSC-like cell populations by MACS. It is possible that this limited “stemness” in chondrocytes, expanded for use in ACI, may instead be beneficial as it allows re-differentiation under appropriate conditions despite prolonged times in culture.

## Introduction

Mesenchymal stem cells (MSCs), also referred to as mesenchymal stromal cells, are undifferentiated cells present in various adult tissues. The multi-potent differentiation capacity of human MSCs was first described by Pittenger *et al*. at the turn of the 20th century [1]. MSCs represent a promising candidate cell population in regenerative medicine and published reports of their application in numerous cases demonstrate their therapeutic potential in the regeneration of all skeletal tissues, as well as of soft tissue organs. One particular advantage of MSCs is that they can be used when cells from the target tissue for regeneration are not available for developing cellular therapy. This is the case in intervertebral disc degeneration and MSC therapy represents a promising tool.

Cartilage and cartilaginous tissues are predominant in regenerative medicine due to the fact that they comprise a simple, homogenous tissue involving only one cell type, the chondrocyte. Consequently, autologous chondrocyte implantation (ACI) became an established cell-based medical procedure for repairing cartilage defects, and, like bone marrow transplantation, it obviously represents a key technology in regenerative medicine. Recent reports have shown that MSCs or undifferentiated progenitor cells are present in cartilage and intervertebral disc tissue [2-8]. Evidently, cartilage contains not only functionally differentiated cells (chondrocytes) but also undifferentiated progenitors or stromal stem cells. This raises the question about whether ACI should still be considered a chondrocyte implantation or whether it is more correctly a stem cell therapy.

The discovery of MSC-like cells in cartilage tissue is significant because it questions the prevailing view that MSC isolated from bone marrow or any other organ or tissue derived essentially from a perivascular niche [9-12]. The cellular component of the perivascular niche that functions as a stem cell in the postnatal organism is the pericyte [9,13]. Once this concept was established, a search began for perivascular niches harboring MSCs and published reports described such niches in several tissues, including human brain tissue [14,15]. Articular cartilage, however, is an avascular tissue devoid of blood vessels (in healthy tissue), a fact that raises the question about the cellular nature and function of MSC-like cells within cartilaginous tissues.

The source of cells for ACI is cartilage [16] or, for disc repair, is disc (prolapse) tissue [17], with the cells usually being expanded *in vitro* and subsequently re-implanted. Alterations in cell properties may occur during *in vitro* manipulation. Expansion may favor particular cell types, and, in terms of chondrocytes, this *in vitro* expansion has historically been described as progressive, and at least partly irreversible, de-differentiation and cellular ageing [18,19]. Changes occur as early as in the first passage [20]. When incubated in three-dimensional constructs, cells may regain their chondrocytic phenotype [21]. However, beyond a certain number of cell doublings or passages, this phenotypic loss is apparently irreversible [22,23]. Pelttari *et al*. showed that chondrocytes which underwent more than six population doublings *in vitro* lost the capacity to form stable ectopic cartilage [24].

On the other hand, this phenomenon may also be described as the regression towards an undifferentiated cell type with higher plasticity which, however, shows a need for specific induction of the cartilage phenotype. Up-regulation of markers regarded as distinctive for MSCs (CD10, CD90, CD105, and CD166) on articular chondrocytes monolayer cultures supports the theory of a reversion to a primitive phenotype [25]. The existence of chondrocyte subpopulations with phenotypic plasticity, that are capable of generating a chondrogenic, adipogenic, and osteogenic lineage, has been reported by several authors [8,26-29].

From a regulatory perspective, it is essential to clarify these cell biological aspects of ACI, particularly in view of future MSC applications in cartilage and disc repair. The aim of the present study was to evaluate the “stem cell” features or “stemness” of chondrocytes populations and identify whether they are advantageous or not within the context of ACI.

To address this issue, the MSC sub-population hypothesis was tested by means of selective enrichment or depletion of cells presenting MSC antigens, using MACS technology, from freshly-isolated primary cultured cells. Subsequently prolonged expansion was done and an analysis of the differentiation capacity followed each stage. The influence of culture medium as a trigger for selection towards a single cell type was also addressed.

MSC surface antigens as detected by monoclonal antibodies (mAb) clone W5C5 (alias SUSD2, shushi domain protein 2) or W8B2 (alias MSC antigen-1 (MSCA-1) or tissue non-specific alkaline phosphatase (TNAP)), known to correlate with specific phenotypic skeletal characteristics, have been used to generate subpopulations. It has been suggested that MSCA-1+/CD56+ MSCs are an attractive starting population for ACI because differentiation experiments had shown that chondrocytes were predominantly derived from this subset [30]. However, the MSCA-1+ fraction of human jaw periosteum-derived cells showed a significantly higher osteogenic capacity than the negative fraction [31]. The antigen recognized by the W5C5 antibody is SUSD2 (Sivasubramaniyan, *et al*.: Prospective isolation of mesenchymal stem cells from human bone marrow using novel antibodies directed against Sushi domain containing protein 2 (SUSD2), submitted); the antibody describes distinct MSC subpopulations and can be used for the prospective isolation of highly-purified bone marrow MSCs [32]. Very recently, W5C5 epitope /SUSD2 was identified as a single marker capable of purifying endometrial MSCs, possessing MSC properties and reconstituting endometrial tissue *in vitro*[33].

We, therefore, investigated whether the selection of cartilage-derived cells by MSC-specific mAbs yielded a chondroid precursor cell type or multi-potent MSC-like cells. We hypothesized that the results would provide evidence about whether *in vitro* expanded chondrocyte cultures are associated with a risk of adverse adipose or osseous differentiation or not, especially after enrichment of putative MSC-like cell populations.

## Materials and methods

### Articular chondrocyte and intervertebral disc cell isolation and culture

Cell isolation and culture were performed as described in [34,35].

Briefly, macroscopically normal appearing cartilage was carefully harvested from the tibial plateau and from the condyles. Tissue samples were washed in phosphate buffered saline (PBS; BioWhittaker; Verviers, Belgium) and then minced. Extracellular matrix was enzymatically degraded overnight using collagenase B (Roche; Mannheim, Germany) and hyaluronidase (Serva; Heidelberg, Germany). Isolated cells were filtered through a cell strainer and after centrifugation the cells were resuspended and used for MACS separation (see below) or expanded in primary culture. To this end, 0.5 x 10^6^ cells were plated in a 75 cm^2^ cell culture flask in DMEM/Ham’s F12 (Lonza; Cologne, Germany) chondrocyte culture medium (CM) supplemented with 10% FCS or human AB serum (HS; IKET; UKT, Tuebingen, Germany) as indicated, 150 μM ascorbic acid-2-phosphate (Sigma-Aldrich; Munich, Germany), 100 U/ml penicillin, 100 μg/ml streptomycin (PAA; Pasching, Austria). The culture was kept at 37°C in humidified atmosphere and 5% CO_2_. The cells were harvested at 80 - 90% confluence by trypsin-EDTA (Lonza; Cologne, Germany) treatment. The harvested cells (“P0 cells”) were used for MACS separation. All human tissues were obtained from the BG Trauma Center in Tuebingen (approved by the local ethics committee and informed consent from all individuals). Details for donors and cell culture conditions are summarized in Additional file 1: Table S1.

### Cell surface antigen profile

To investigate if chondrocytes and disc cells express typical MSC surface markers flow cytometry was performed. Therefore phenotype profiling was performed with P0 cells. The cells were detached, washed, incubated on ice with phycoerythrin (PE)-conjugated monoclonal antibodies directed against CD34, CD45, CD49e, CD73, CD90, CD105, CD140b, CD166, CD271, W8B2 (MSCA-1), W5C5 (SUSD2), according manufacturer’s instructions (for further information see Additional file 1: Table S2) and analyzed by flow cytometry (Cytomix FC500; Beckman Coulter; Krefeld, Germany). Additionally for comparison with P0 profiles, surface antigen profiles of chondrocyte subpopulations after expansion > 8 population doublings (pds) were performed using the same protocol.

### Enrichment of cells using W5C5- and W8B2-coupled magnetic microbeads

W5C5^+^ and W8B2^+^ cell populations were enriched by magnetic separation using the MACS® technology. The magnetic labeling and the magnetic separation were performed according to the supplyers protocol # 130-093-583 for the anti-MSCA-1 (W8B2, Microbead Kit, Miltenyi Biotech, Bergisch Gladbach, Germany). Briefly, human chondrocytes or human disc cells directly after enzymatic digestion or from first passage (P0) were attached to anti-W5C5 or anti-W8B2 microbeads, respectively, for 15 min. at 4°C. Magnetic separation resulted in three cell populations: the unsorted whole cells (unsorted), the positively enriched population (pos. +) and the depleted (neg. -) fraction. To analyze the quality of the separation flow cytometry (see above) based on the same antibodies was performed after MACS separation.

### Gene expression analysis

The gene expression analysis was performed in parts according to MIQE guidelines [36]. Total RNA was extracted using the RNeasy mini kit plus DNase I digestion according to the manufacturer’s instructions (Qiagen; Hilden, Germany). RNA purity was determined by photometric measurement of the 260 nm/280 nm ratio of the sample using a Bio Photometer (Eppendorf #613125116, Hamburg, Germany). Complementary DNA (cDNA) was obtained by reverse transcription of up to 1 μg total RNA using the Reverse Transcriptase Core kit (Eurogentec; Cologne, Germany) with EuroScript reverse transcriptase (Moloney Murine leukemia virus rev. transcriptase, 50 U/μl) and oligo-dT primers. Reverse transcription was performed in a total volume of 50 μl at 48°C for 30 min. in a thermocycler (Whatman Biometra; Goettingen, Germany).

Gene expression was analyzed by semi-quantitative real-time PCR using an Applied Biosystems 7500 Fast Real-Time PCR System as described in [34]. The qPCR mastermix plus for SYBR green I (low ROX) kit from Eurogentec was used. Primers were designed with the primer express 2.0 software (Applied Biosciences; Darmstadt, Germany), except primers for type II collagen (Col2A1) [37], and were obtained from BIOTEZ. Sequences of all primers used are summarized in Additional file 1: Table S3. Glyceraldehyde-3-phosphate dehydrogenase (GAPDH) was used as reference gene. Quantification cycles (Cq values) were determined for each gene using Sequence Detection System software (Applied Biosystems) [38]. PCR efficiencies (E) were calculated for each primer pair using a calibration curve. Relative gene expression was calculated according to the following equation:

$$\mathrm{relative}\;\mathrm{mRNA}\;\mathrm{expression}=\frac{E^{\mathrm{Cq}\;\left(\mathrm{reference}\;\mathrm{gene}\right)}}{E^{\mathrm{Cq}\;\left(\mathrm{marker}\;\mathrm{gene}\right)}}\text{.}$$

### Analysis of the chondrogenic state of differentiation after sorting

Five x 10^5^ cells were embedded into 500 μl human serum albumin – hyaluronan (HSA-HA) hydrogel directly after the MACS separation. HSA-HA preparation and cell embedding in hydrogels are described in [34]. The gels were overlaid with either 1.5 ml chondrocyte culture medium (CM) or chondrogenic induction medium (DMEM high glucose, 4.5 g/l (Invitrogen), 100 U/ml penicillin, 100 μg/ml streptomycin, 4 mM L-glutamine, 1 mM sodium-pyruvate, 170 μM ascorbic acid 2-phosphate, 0.1 μM dexamethasone, 1 × ITS supplement, 0.35 mM L-proline (all from Sigma-Aldrich) and 10 ng/ml TGF-β_3_ (Miltenyi Biotech) and cultured for six weeks. Medium was changed twice a week for a total culture time of six weeks. Cell culture supernatants were collected for biochemical analysis. Subsequently, the gels were either frozen for biochemical analysis, or digested with proteinase K (3 mg/ml; Sigma-Aldrich) for about 15 min. at 37°C to recover the cells for gene expression analysis. The digest was centrifuged and the cell pellet lysed in 350 μl lysis buffer (RLT-buffer, Qiagen; Hilden, Germany).

### Biochemical analysis

To analyze the extracellular matrix production the glycosaminoglycan (GAG) content was determined in the digested hydrogels and in the supernatants. Hydrogels were digested with 1 mg/ml papain (Sigma-Aldrich) in 0.1 M Na-acetate, 0.01 M L-cysteine, 0.05 M Na2-EDTA, and 0.2 M NaCl (pH 6.0) at 60°C overnight and stored at -80°C. Culture supernatants were used without digestion. Supernatants were freeze-dried and stored at -80°C. For measurement the supernatants were re-constituted with H_2_0 and thereby concentrated by a factor of 5. The GAG content was measured by the dimethylmethyleneblue (DMB) assay, including guanidinium hydrochloride in the protocol and using chondroitin-4-sulfate (Sigma-Aldrich) as a standard [39]. Proteoglycan content was normalized to DNA content and expressed as μg GAG/ μg DNA. The DNA content in the hydrogels was determined using the PicoGreen fluorescent dye (Molecular Probes/Invitrogen). Standard curves were generated at the time of each measurement using known concentrations of phage lambda DNA (Eppendorf; Hamburg, Germany). DNA content was expressed as μg DNA.

### Analysis of replicative capacity - cell growth curves

Cells were plated at a starting cell density (6.7 x 10^3^ cells/cm^2^), cultivated to reach confluence (80 – 90%), and harvested using trypsin/EDTA. The cell number was determined. The number of population doublings (pds) was calculated using the following equation: log (cell number at harvest/seeding cell number)/log2. A cell aliquot was used for gene expression analysis. Cells were expanded for several passages under identical conditions until they underwent at least 8 to 10 pds after MACS separation. Then the cells were used for differentiation experiments. Two cultures were additionally expanded in mesenchymal stem cell growth medium (MSCGM, Promocell; Heidelberg, Germany) to test the influence of the medium. These cultures are marked in the results by the suffix “MSCGM” to the culture number.

Growth curves were calculated as a function of population doublings over the cultivation time. To describe changes in the cellular phenotype dependent from the population doublings, the expression of collagen type I α2 (COL1a2), collagen type II α21 (COL2a1), SRY (sex determining region Y)-box 9 (SOX9), and core-binding factor subunit alpha-1 (CBFA1, also known as Runt-related transcription factor 2 (RUNX2)) was analyzed directly after MACS, after 4 +/- 1 pds, and after > 8 pds.

### In vitro differentiation protocols and related read-out assays

To analyze the differentiation potential, cells were induced to differentiate in the adipogenic, osteogenic and chondrogenic lineage.

### Adipogenic induction

For adipogenic induction a commercially available adipogenic differentiation medium was used (Promocell # C-28011) and the differentiation performed according to the manufacturer’s instructions. Briefly, 3.15 x 10^4^ cells/cm^2^ were plated in a tissue culture plate using MSC Growth Medium. Cells were allowed to reach 80 – 90% confluence, and then the medium was exchanged for MSC adipogenic differentiation medium. Controls were maintained in MSC growth medium. After induction the cells were cultured for three weeks, medium was changed twice a week. At the end of the culture period cells cultured in 24-well plates for gene expression analysis were lysed directly with 500 μl lysis buffer per well (RLT buffer, Quiagen; Hilden, Germany). Cells cultured in 96-well plates were used for Oilred O staining and were processed as described below.

#### Gene expression

The induction of adipogenic specific genes, Peroxisome proliferator-activated receptor gamma (PPARγ2) and adiponectin, was analyzed by semi-quantitative real-time PCR in cell cultures after adipogenic induction and compared to controls.

#### Oil red O staining

To localize lipid droplets, cell layers were fixed in 4% paraformaldehyde, incubated in 60% n-propanol, and then stained in Oil Red O solution for 10 minutes. Cells were washed and cell nuclei counterstained with Mayers haemalaun solution for 3–5 minutes. Cell layers were rinsed with tap water to develop the blue nuclei staining and then microscopically observed.

### Osteogenic induction

For osteogenic induction a commercially available osteogenic differentiation medium was used (Promocell # C-28013) and the differentiation performed according to the manufacturer’s instructions and as described under “adipogenic induction” with the exception that cells were allowed to reach > 100% confluence before the medium was exchanged for osteogenic differentiation medium. Cells cultured in 96-well plates were used for the determination of alkaline phosphatase (ALP) activity and Alizarin Red staining and were processed as described below.

#### Gene expression

The induction of osteogenic specific genes, CBFA1 and ALP, was analyzed as described above.

#### Quantitative assay of ALP activity

ALP activity was determined using p-nitrophenyl phosphate as substrate. Cell cultures (triplicates in 96-well plates) were washed and incubated with 100 μl 0.01% Triton X-100 for 30 min. at 4°C. 50 μl of the lysate was transferred into another 96-well plate and mixed with 50 μl of 0.01% Triton X-100. Then 200 μl p-nitrophenyl phosphate were added per well and the plate was incubated for ≥ 30 min. at RT. The absorbance at 405 nm was measured using a Pherastar plate reader (BMG Labtech, Offenburg, Germany). Serial dilutions of p-nitrophenol were used for the standard curve. The ALP activity was expressed as substrate conversion per time [pmol/min.] and was normalized to the DNA content [μg] of the corresponding well.

#### Alizarin red staining

To identify mineral deposits by Alizarin Red S, the cell monolayers were washed and fixed with ice-cold 50% methanol, 50% acetone and were then air dried. After washing the cells were stained with 40 mM Alizarin Red S (pH 4.0) for 20 minutes. After extensive washing the cell layer was dehydrated with ice-cold 100% ethanol and air-dried before microscopically examination.

### Chondrogenic induction

Chondrogenic differentiation potential of the cells was analyzed in HSA-HA hydrogels. HSA-HA preparation and cell embedding into hydrogels are described in [34]. Five x 10^5^ cells were embedded into 500 μl hydrogel. After a few minutes of polymerization the gels were cultured in 1.5 ml chondrogenic induction medium (see above). Controls were cultured in the same medium without TGF-β_3_. Hydrogels were cultured for four weeks; medium was changed twice a week. Cell culture supernatants were collected for biochemical analysis. After four weeks the gels were frozen for biochemical analysis and digested with proteinase K to recover the cells for gene expression analysis respectively.

#### Gene expression

The induction of chondrogenic specific genes SOX9, COL2a1, and aggrecan (ACAN) was analyzed as described above.

### Statistics

Gene expression data were used as logarithmic values to obtain data which follow Gaussian distribution.

**Figure 1:**
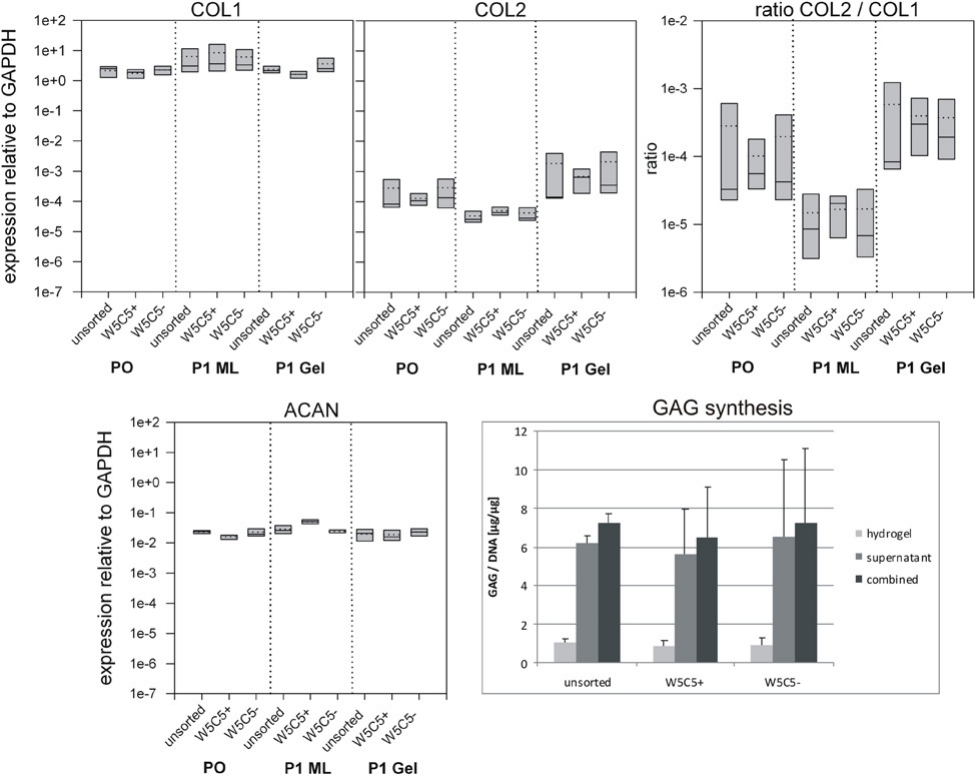
**Differentiation of chondrocytes and sorted sub-populations.** Expression of mRNA in primary expanded human chondroyctes after MACS separation (P0), and subsequent passage in monolayer (P1 ML) or in hydrogel culture (P1 Gel). The P1 ML cells were cultured in chondrocyte medium (CM) until they had reached approx. 80% confluence (about 1 week), hydrogels were maintained in CM for 6 weeks. The expression of collagen type I (COL1), type II (COL2), and aggrecan (ACAN) was analyzed and the ratio of COL2/COL1 was calculated as a measure to describe the chondrogenic phenotype of the cells as indicated. Box plots represent 25% / 75% percentiles, mean values (dotted lines) and median values (hairlines). Lower right: the cumulated glycosaminoglycan production from cells in hydrogel culture was determined. GAG content was measured in the hydrogel and in the combined medium supernatant of the six-week cultures and normalized to the corresponding DNA content in the hydrogel. Mean values and standard deviation are shown (n = 3 cultures).

**Figure 2:**
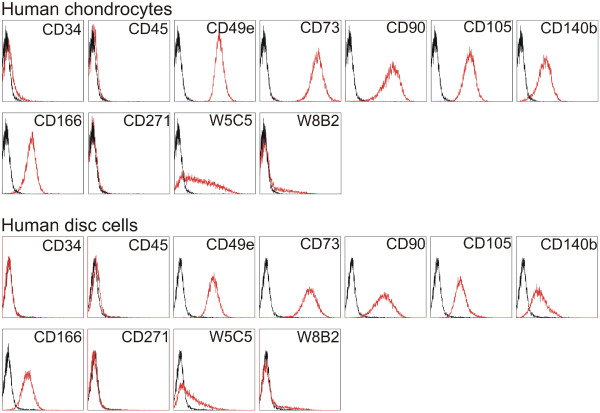
**Cell surface antigen expression of selected markers on cartilage cell populations.** Human chondrocytes (P0), and human disc cells (P0) were stained with the indicated phycoerythrin-conjugated monoclonal antibodies. The black lines show the fluorescence intensity of the IgG negative control antibody, whereas the intensity of the antibody of interest is shown in red. Representative analyses are shown.

**Figure 3:**
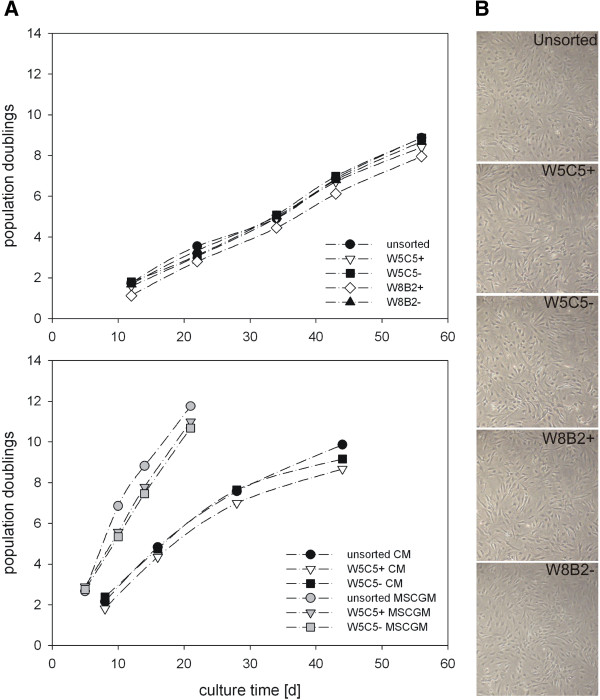
**Cell growth and morphology of chondrocytes and sub-populations. A**) Unsorted chondrocytes and W5C5 or W8B2 enriched (+) or depleted (-) sub-populations of the same culture (Cho4) were expanded over several passages. Population doublings and culture time are presented (top). Chondrocytes (Cho8) and W5C5 ± sub-populations are cultured in chondrocyte culture medium (CM) or MSC growth medium (MSCGM). Growth curves are shown (bottom). **B**) Cell morphology of different sub-populations. (Due to the low expression of W8B2 on chondrocytes, the number of sorted cells was too low for a complete set of experiments).

**Figure 4:**
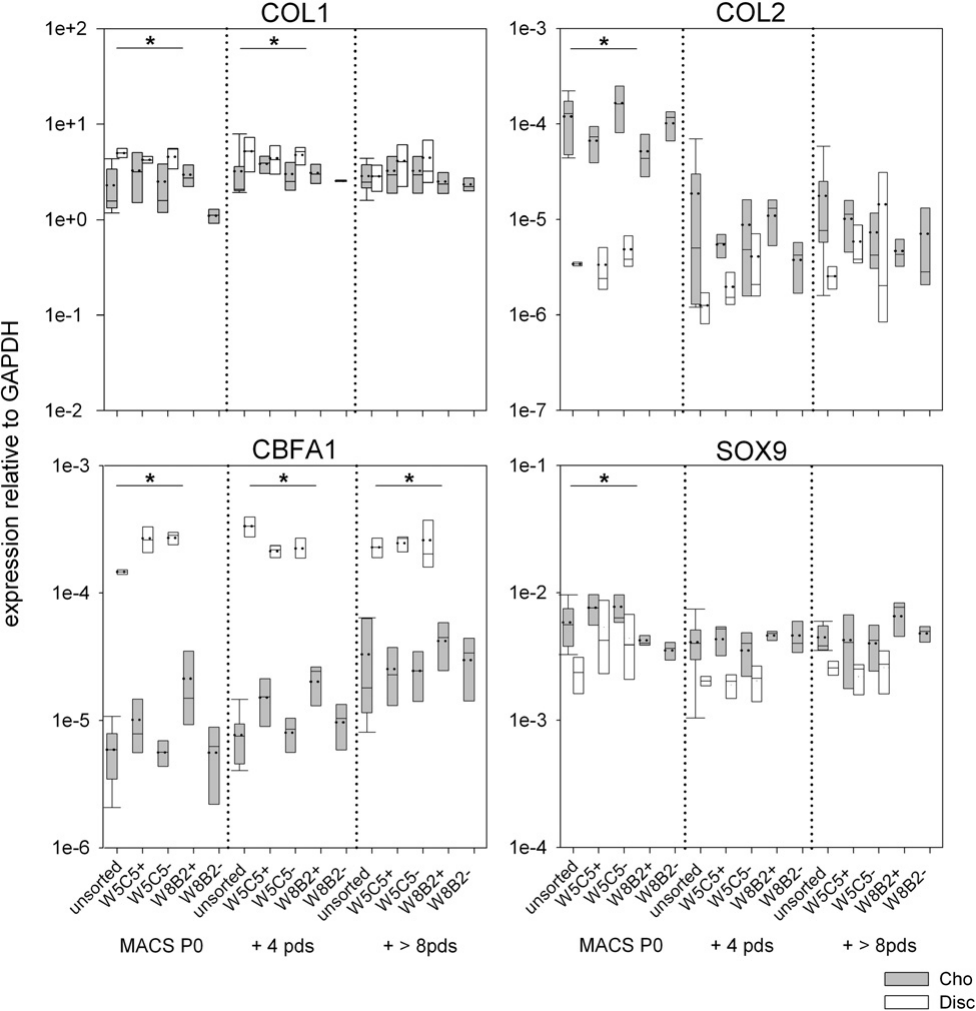
**Gene expression of chondrocytic sub-populations dependent on the population doublings.** Primary expanded chondrocytes and disc cells were MACS separated and expanded in monolayer cultures over several passages. Gene expression was determined at the end of the first passage after MACS sorting (MACS P0), when cells had undergone 4 ± 1 pds after MACS, and after cells had undergone more than 8 pds after MACS. Expression of collagen type I (COL1), collagen type II (COL2), and the transcription factors SRY (sex determining region Y)-box 9 (SOX9) and core-binding factor subunit alpha-1 (CBFA1) were analyzed and expressed relative to the reference gene GAPDH. *: significant difference (p < 0.05) between all chondrocyte populations compared to all disc populations. (For mean expression values see Additional file 1: Table S4).

**Figure 5:**
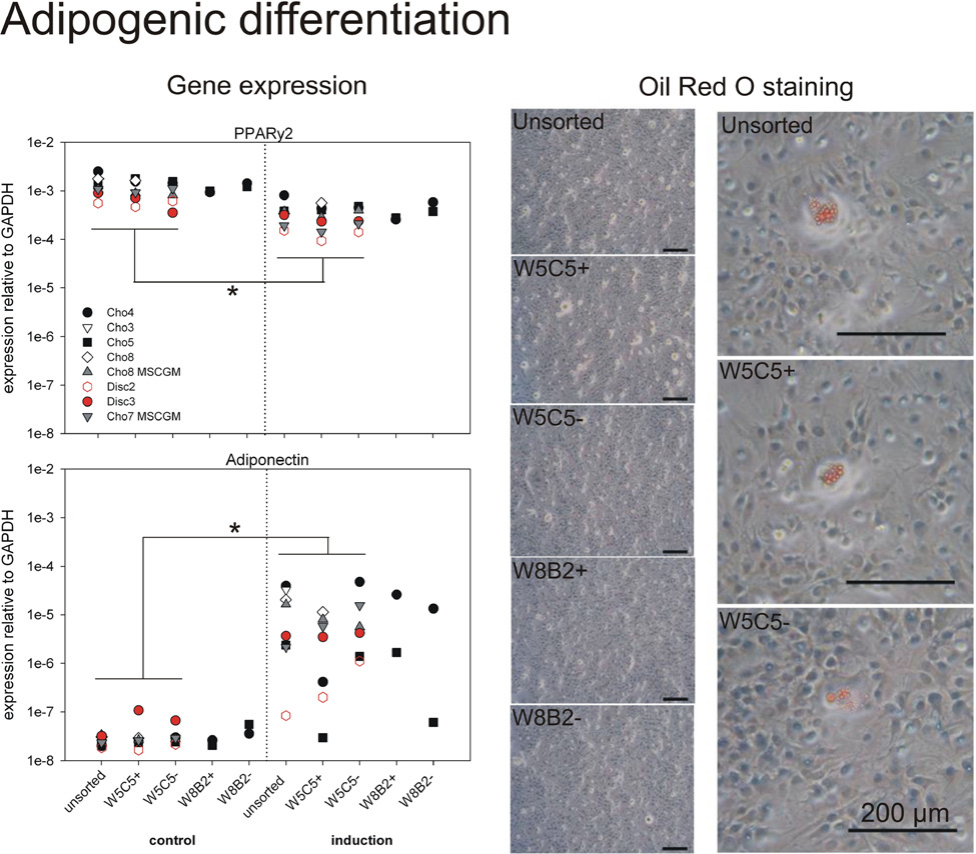
**Adipogenic differentiation of sub-populations after > 8 pds.** Adipogenic differentiation was induced using adipogenic induction medium, control cells were cultured in MSC growth medium (MSCGM). Three weeks after induction gene expression of the adipogenic markers, the transcription factor peroxisome proliferator-activated receptor γ 2 (PPARγ2) and adiponectin was analyzed. Expression relative to GAPDH is presented for each individual culture (left). In parallel, lipid droplets were visualized by Oil Red O staining (bars = 200 μm). Representative pictures of one culture after induction are shown. Only very sporadically were positive cells found (pictures with higher magnification on the right). None of the control cultures was positive. *: significant difference (p < 0.05) between all control cultures compared to all cultures under induction conditions. (For mean expression values see Additional file 1: Table S5).

**Figure 6:**
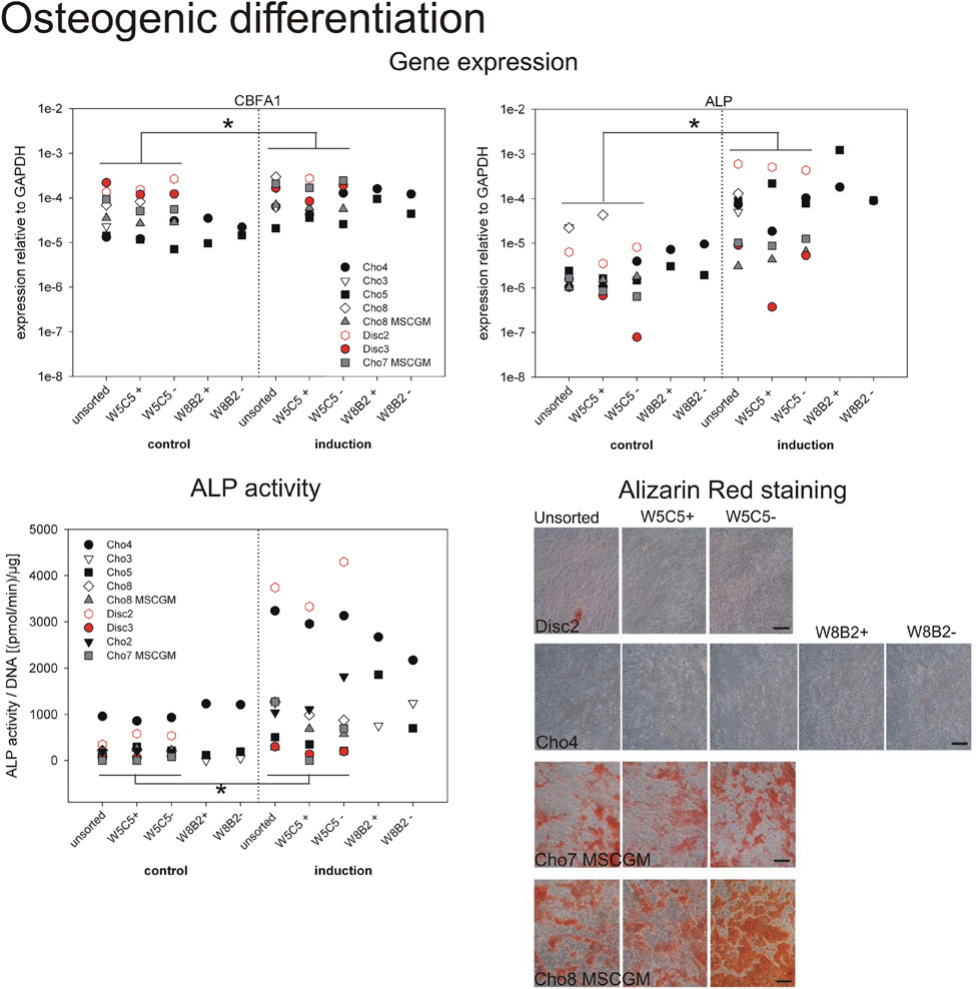
**Osteogenic differentiation of sub-populations after > 8 pds.** Osteogenic differentiation was induced using osteogenic induction medium, control cells were cultured in MSC growth medium (MSCGM). Three weeks after induction gene expression of the osteogenic markers, the transcription factor core-binding factor subunit alpha-1 (CBFA1) and alkaline phosphatase (ALP) were analyzed. Expression relative to GAPDH is presented for each individual culture (top). ALP activity was determined in the cell lysates at the end of the culture and was normalized to the corresponding DNA content in the well. In parallel, mineralization was visualized by Alizarin Red staining (bar = 200 μm). Representative pictures of four induced cultures are shown. None of the control cultures was positive. *: significant difference (p < 0.05) between all control cultures compared to all cultures under induction conditions. (For mean expression values see Additional file 1: Table S6).

**Figure 7:**
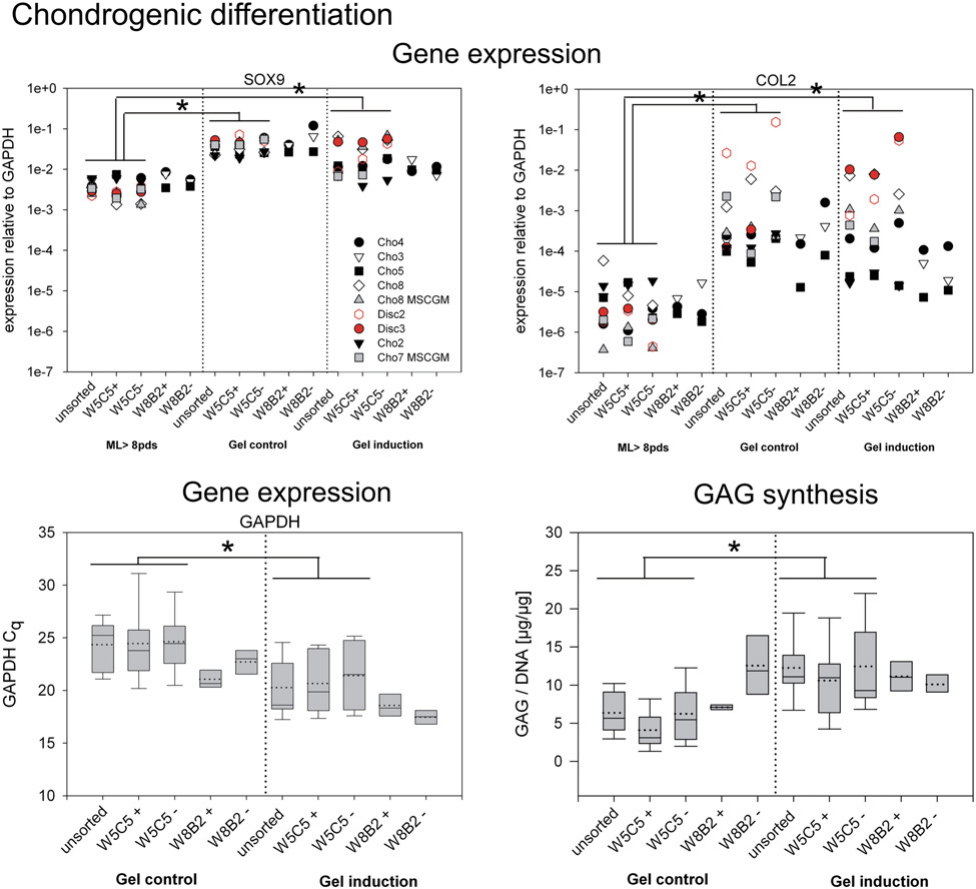
**Chondrogenic differentiation of sub-populations after > 8 pds.** Chondrogenic differentiation was performed in hydrogels and induced with TGFβ_3_, control cells were cultured in hydrogels in the same medium without TGFβ_3_. Four weeks after induction gene expression of the chondrogenic markers, the transcription factor SRY (sex determining region Y)-box 9 (SOX9), collagen type II (COL2) was analyzed and compared to the expression of the cells before being embedded into the hydrogels (ML > 8pds). Expression relative to GAPDH is shown for each individual culture (top). Quantification cycles (C_q_) for GAPDH expression are shown in the box plot. GAG synthesis (GAG content of the hydrogel + content in the cumulated culture supernatants) is presented as normalized to the DNA content (lower right). Boxes represent 25% / 75% percentiles, whisker 5% / 95% percentiles, mean (dotted line) and median (solid line) values. *: significant difference (p < 0.05) between all ML > 8 pds cultures compared to all gel control cultures and compared to gel cultures under induction conditions. (For mean expression values see Additional file 1: Tables S7 and S8).

**Figure 8:**
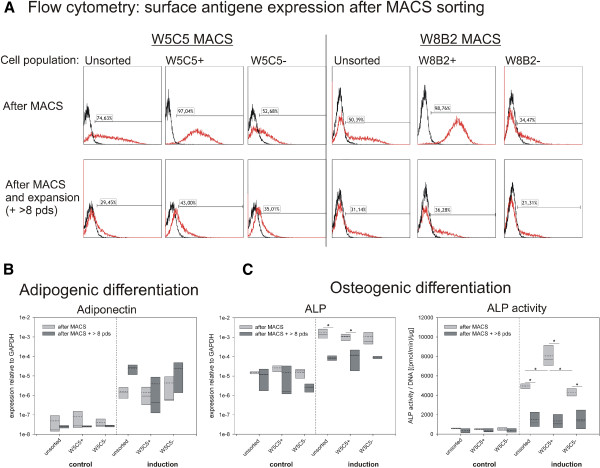
**Comparison of chondrocyte sub-populations as a function of population doublings.** Cells were analyzed at the end of the first passage after MACS and subsequent expansion until cells had undergone more than 8 population doublings (pds). **A**) Flow cytometry analysis with percentage of W5C5 positive cells after W5C5 MACS sorting and W8B2 positive cells after W8B2 MACS sorting. Representative examples are shown. **B**) Adipogenic differentiation of subpopulations. Adiponectin expression relative to GAPDH. **C**) Osteogenic differentiation. ALP gene expression relative to GAPDH and ALP enzyme activity were presented. Boxes represent 25% / 75% percentiles, whisker 5% / 95% percentiles, mean (dotted line) and median (solid line) values. (n = 3 donors after MACS, n = 4 donors + > 8pds). *: significant differences (p < 0.05) between cells differentiated directly after MACS and after expansion, and between subpopulations under the same experimental conditions. Significant differences existed in all cases between the control and the induction conditions (not specifically indicated).

Statistical analysis was not necessary for differentiation of chondrocytes (Figure 1), cell surface antigen expression (Figure 2), and cell growth and morpholgy (Figure 3). For results presented in Figure 4: One Way Analysis of Variance (ANOVA) was performed if data passed normality test (Tukey-Kramer Multiple Comparisons Tests). If data did not pass the normality test nonparametric ANOVA was performed (Kruskal-Wallis Test). For results presented in Figures 5, 6, 7 and 8: Two hypotheses were tested in the differentiation assays: 1) “There is a significant difference between control and induction conditions”. To confirm the first hypothesis an unpaired t-test comparing all control samples with all induced samples was performed. When data did not pass the normality test, a Mann–Whitney test was used. 2) “There are differences between the subpopulations/subgroups under the same experimental conditions”. To confirm the second hypothesis two ANOVAs were performed comparing unsorted, W5C5+ and W5C5- populations either under control or under induction conditions.

Due to the small number of W8B2 +/- samples, they were excluded from statistical analysis. In “Results”, the use of the term “significant” relates to a p < 0.05 level of significance.

## Results

### Feasibility of MACS sorting of freshly isolated or pre-cultured primary chondrocytes and disc cells

To prove the feasibility of the MACS technique of chondrocytes and disc cells, directly from tissue digests or after expansion in primary culture, an experimental set-up was installed as described below (Additional file 2: Figure S1). Cartilage or disc tissue was enzymatically digested and the isolated cells either used directly for MACS sorting or seeded into a primary culture. The primary cells were expanded until they reached 80 – 90% confluency and were then analyzed by MACS. The obtained sub-populations were tested in a three-dimensional hydrogel which is already being used clinically (http://www.clinicaltrials.gov; identifier:NCT01640457).

### MACS with freshly isolated chondrocytes

In the first set of experiments, the magnetic separation of freshly-isolated chondrocytes after overnight enzymatic digestion was examined. As the MACS technology is available for clinical use, it is important to clarify whether the cell sorting process could be embedded into a clinical set-up, i.e. as a one-step operating procedure. All attempts to separate freshly-isolated cells by MACS have not been successful: the separation columns clogged after the cell suspension was applied to the separation columns, and it was not possible to carry out the essential washing steps. Instead, the cells had to be pushed out by means of a plunger and then layered on to a new column. Following this, it was occasionally possible to proceed successfully and to carry out the separation. The recovery rate was only about 40% and the percentage of positive cells was below 10% of all cells. In the majority of cases, however, successful separation could not be achieved. Therefore, the magnetic separation of freshly-isolated chondrocytes was not considered to be a reliable and efficient method.

### MACS with primary expanded chondrocytes / disc cells

Consequently, the magnetic separation was carried out using cells from primary expanded cultures. The results of the recovery, efficiency and quality of the separation are summarized in Table 1.

**Table 1:** MACS technical data from primary expanded chondrocytes or disc cells (“P0 cells”)

**W5C5 sorting**								
	**Cell numbers [ x 10**^**6**^**]**					**Analytical flow cytometry: % W5C5 positive cells in**		
**Cell type**	**Initial cells**	**Bound fraction**	**Flowthrough fraction**	**Total recovery [%]**	**% Bound cells from initial cells**	**Bound fraction**	**Flowthrough fraction**	**Unsorted fraction**
**chondrocytes**								
Cho2	8.9	1.6	4.5	68.0	18.0	n.d.	n.d.	n.d.
Cho4	16.2	4.0	5.0	55.7	24.8	95.9	53.8	55.2
Cho5	8.8	2.2	4.7	78.4	24.5	86.6	47.8	74.7
Cho6	10.0	3.2	4.3	75.0	32.0	87.4	26.2	51.2
Cho7	37.2	4.8	30.5	94.9	12.9	75.4	12.0	24.9
Cho8	8.8	1.3	3.3	52.2	14.9	90.6	28.0	42.8
Cho9	12.8	2.9	6.2	71.1	22.7	78.5	24.9	41.7
Cho10	24.1	6.8	9.2	66.3	28.1	92.8	38.4	63.0
Cho11	13.1	2.0	6.5	64.7	14.9	94.5	30.1	43.7
Cho12	8.4	0.5	4.7	62.1	6.0	70.2	37.4	31.1
Cho13	19.3	4.4	8.7	68.0	22.7	97.0	52.7	74.6
Cho14	21.9	6.7	5.5	55.7	30.6	97.2	69.2	89.3
Cho15	9.0	0.9	3.7	51.4	10.4	95.1	32.2	42.2
mean	15.3	3.2	7.4	66.4	20.2	88.4	37.7	52.9
sd	8.5	2.1	7.2	12.0	8.0	9.1	15.7	19.2
**disc cells**								
Disc1	n.d.	0.6	6.4	n.d.	n.d.	n.d.	n.d.	n.d.
Disc2	7.6	1.2	1.6	37.0	16.1	n.d.	n.d.	n.d.
Disc3	13.7	1.5	4.9	46.3	10.6	98.1	45.1	49.4
mean	10.7	1.1	4.3	41.6	13.3	98.1	45.1	49.4
sd	4.3	0.4	2.5	6.6	3.9			
**W8B2 sorting**								
	**Cell numbers [ x 10**^**6**^**]**					**Analytical flow cytometry: % W8B2 positive cells in**		
**Cell type**	**Initial cells**	**Bound fraction**	**Flowthrough fraction**	**Total recovery [%]**	**% Bound cells from initial cells**	**Bound fraction**	**Flowthrough fraction**	**Unsorted fraction**
**chondrocytes**								
Cho3	9.5	0.6	5.8	67.3	5.8	n.d.	n.d.	n.d.
Cho4	16.2	0.7	7.2	48.5	4.3	72.8	8.7	5.6
Cho5	8.8	0.7	6.3	79.4	8.2	63.0	11.9	16.8
Cho14	22.4	1.1	7.3	37.4	4.8	98.8	34.5	50.4
mean	14.2	0.8	6.6	58.1	5.8	78.2	18.4	24.3
sd	6.4	0.2	0.7	18.8	1.7	18.5	14.1	23.3

With primary chondrocytes and primary disc cells MACS sorting using W5C5 and W8B2 microbeads was feasible and led to satisfying results. On average, approx. 60% of the cells initially used could be recovered. The percentage of W5C5 positive cells was 20.2% ± 8.0 for chondrocytes and 13.3% ± 3.9 for disc cells. After W8B2 sorting, only 5.8% ± 1.7 positive chondrocytes were obtained: in other words, on average, only 0.8 x 10^6^ cells were in the bound fraction (positive fraction) of a typical cell preparation. However sufficient cell numbers were not obtained and therefore re-differentiation of W8B2 cells in hydrogels was not pursued. Only W5C5-enriched and W5C5-depleted sub-populations were analyzed further.

The efficacy of separation by MACS varied considerably. In the W5C5+ fractions, about 88% of the cells were positive for W5C5 as determined by flow cytometry, but the W5C5- fraction still contained more than 1/3 positive cells. The W8B2+ fraction was even less pure, and only about 78% of the cells were positive when investigated by flow cytometry; with the depleted fraction contained about 18% positive cells. After MACS sorting, the cell cultures showed a high viability and cell attachment was not impaired by the magnetic labeling (data not shown).

### Analysis of the chondrogenic state of differentiation after sorting

To analyze whether the chondrogenic phenotype and differentiation state correlated with the presentation of the W5C5 antigen on chondrocytes, the gene expression of collagen type I and type II, and of aggrecan in cells was analyzed directly after MACS separation (P0), and in passage 1 cells cultured in monolayer or in three-dimensional hydrogel culture (Figure 1). To describe differentiation, the ratio of collagen type II / collagen type I was calculated. Collagen type I and aggrecan were very stably expressed at high levels in all sub-populations, regardless of the monolayer or hydrogel culture. In contrast, collagen type II expression decreased in the P1 monolayer cells (P1 ML) as compared to P0 cells, but the expression increased in the parallel hydrogel culture. In other words, the three-dimensional environment in the hydrogel culture was able to stabilise, and possibly even slightly improve, the chondrogenic phenotype. The COL2 / COL1 ratio of the hydrogel cultures was about 20 times higher than in P1 ML cells. The result was the same for all three sorted cell populations (Figure 1).

To further describe the quality of the hydrogel cultures, the amount of synthesised glycosaminoglycans was determined using a DMB assay. We did not find meaningful differences in the GAG content, either within the hydrogels nor when released into the supernatant during the six weeks of culture (Figure 1, lower right). The unsorted chondrocyte population, as well as the W5C5+ and W5C5- fractions had a cumulative GAG production of about 6 μg GAG per μg DNA, and GAGs were mostly secreted into the culture medium (on average > 85%). The cells synthesized about 1 μg GAG per μg DNA per week.

### MSC surface antigen presentation on chondrocytes and disc cells

The presence of cells displaying some of the typical MSC antigens in cartilage tissue has been already shown [29,40]. Therefore, investigations focused on the MSC surface marker antigens that were also present on our chondrocytes and disc cell populations. The expression of CD34, CD45, CD49e, CD73, CD90, CD105, CD140b, CD166, CD271, W5C5, and W8B2 was analyzed (Figure 2).

Cultured chondrocytes as well as disc cells (P0), have been shown to share common surface antigens with MSCs [41]. Like MSCs, they are positive for CD49e, CD73, CD90, CD105, CD140b, and CD166. Lower expression of W5C5 and W8B2 was found, although a few chondrocytes displayed high intensity (Figure 2; see also Table 1). The cells are negative for CD34 and CD45; this is by definition also a required feature for MSC. As reported for MSC in culture [30,32], CD271 is not expressed on cultured chondrocytes and disc cells.

### Analysis of MSC characteristics of W5C5 and W8B2 positive sub-populations

To investigate whether W5C5 or W8B2 sub-populations isolated from chondrocyte or disc cell cultures show MSC characteristics, their replicative capacity and differentiation potential were analyzed based on the following experimental set-up (Additional file 2: Figure S2).

### Analysis of the replicative capacity of chondrocytic sub-populations dependent on the W5C5 or W8B2 antigen presentation

To investigate whether the expression of W5C5 or W8B2 was related to the replicative properties of cell sub-populations, MACS-separated cell populations were cultured over several passages until they had undergone more than 8 pds and growth curves were calculated. The cell morphology during culture was photo-documented.

Table 2 shows the average culture time necessary to obtain about ten population doublings for the cultures. Chondrocytes needed more than 63 days to reach on average approx. 9.5 pds. They grew at an average growth rate of 0.16 pds/d. The growth rate was relatively constant during the passages, but, for some cultures, growth slowed down during the last passage (Figure 3A). Only minor differences were observed between the different sub-populations and the unsorted whole cell population. In addition, no morphological differences could be seen (Figure 3B). Chondrocytes that were grown in MSC growth medium (MSCGM) were able to divide much faster, and the pds/d increased to about 0.5 pds/d. When compared to articular cartilage chondrocytes, disc cells grew about 2.6-fold faster (keeping in mind that disc cells were maintained in medium + HS and chondrocytes in either FCS or HS).

**Table 2:** Culture time, population doublings (pds), and growth rate of chondrocytic subpopulation after MACS separation

**W5C5 sorting**									
**Cell type /**	**Mean**	**no. of**	**Mean**	**Population doublings (pds) /day**					
**medium**	**culture time**	**passages**	**pds**	**Unsorted**		**W5C5+**		**W5C5-**	
	**[d]**			**Mean**	**sd**	**Mean**	**sd**	**Mean**	**sd**
chondrocytes	63.50	4 - 6	9.47	0.16	0.04	0.15	0.04	0.16	0.04
n = 4									
chondrocytes	24.50	4	11.53	0.51	0.07	0.49	0.05	0.48	0.04
MSCGM; n = 2									
disc cells	25.67	4	11.44	0.42	0.13	0.47	0.12	0.47	0.14
n = 3									
**W8B2 sorting**									
**Cell type /**	**Mean**	**no. of**	**Mean**	**population doublings (pds) /day**					
**medium**	**culture time**	**passages**	**pds**	**Unsorted**		**W8B2+**		**W8B2-**	
	**[d]**			**mean**	**sd**	**mean**	**sd**	**mean**	**sd**
chondrocytes	70.00	4 - 6	10.57	0.15	0.01	0.14	0.01	0.15	0.01
n = 3									

To investigate whether phenotypic alterations or any spontaneous differentiation had occurred during culture, the gene expression of collagen type I and II and of the two transcription factors SOX9 and CBFA1 was analyzed.

We originally did not intend to differentiate between chondrocytes and disc cells; however, as their behavior was significantly different, we have depicted the results for the respective cell type (Figure 4). The expansion of chondrocytes in MSCGM altered the gene expression (see Additional file 1: Table S4). As only minor differences were observed between the mAb enriched sub-populations, we combined all sub-populations for statistical analyses and compared the three groups “chondrocytes”, “disc cells”, and “chondrocytes MSCGM” using ANOVA (data for MSCGM were not shown in Figure 4, only in Additional file 1: Table S4). However, W8B2 +/- sub-populations were excluded from the statistical analysis due to the small sample size.

On first view, a very uniform expression of collagen type I and SOX9 in chondrocytes and disc cells was observed (Figure 4). These genes were expressed at comparable levels, regardless of the population doublings the cells had undergone. Collagen type I expression varied only in the range of one decade, SOX9 actually only by a factor of 4. COL1 expression was slightly but significantly (p < 0.05) higher in disc cells compared to chondrocytes in P0 after MACS and, in cells cultured for additional 4 pds. SOX9 expression was significantly lower in PO discs cells after MACS. Collagen type II expression also differed significantly in PO cells. Expression was 30-fold higher in chondrocytes compared to disc cells. In chondrocytes, the collagen type II expression decreased with increasing pds, but remained more or less constant in disc cells. These results demonstrate once again that the progressive de-differentiation process of chondrocytes results in a lower ratio of collagen type II to collagen type I. It also shows that, with increasing pds, the cells became increasingly homogenous. In contrast, disc cells remained phenotypically more stable.

CBFA1 expression in disc cell was significantly higher than in chondrocytes, regardless of the stage of the expansion process. Disc cells showed an almost constant mean expression ranging from 2.39 to 2.49 x 10^-4^ relative to GAPDH independent from the expansion state. In chondrocytes the CBFA1 expression increased slightly, from a mean value of 7.28 x 10^-6^ in PO cells to a 2.52 x 10^-5^ after they underwent additional > 8 pds.

In terms of the presentation of W5C5 or W8B2 antigens, we found no obvious changes, neither for the replicative capacity, nor for gene expression (See Additional file 1: Table S4).

### Analysis of the differentiation capacity of chondrocytic subpopulations depending on W5C5 or W8B2 antigen presentation

Because MSCs maintain their differentiation capacity for several population doublings *in vitro* whereas differentiated chondrocytes lose their re-differentiation potential after only a few population doublings [22-24], we used cells for the differentiation assays which had undergone at least 8 pds to create most stringent conditions for detecting true MSCs in the chondrocytic cell types. Adipogenic, osteogenic and chondrogenic differentiation was induced using standard protocols and media. Data of the gene expression analysis are presented as scatter plots for each culture in order to be able to single out conspicuous cultures. There was no difference in the cellular differentiation patterns in cells obtained by the two baseline conditions with HS and FCS despite modest differences in proliferation rates and phenotypic alterations during expansion. Therefore, the two standard conditions were kept together and only discriminated against MSCGM.

### Adipogenic differentiation

To determine the adipogenic differentiation potential of chondrocytic sub-populations, gene expression of the transcription factor peroxisome proliferator-activated receptor γ 2 (PPARγ2) and adiponectin, a peptide hormone exclusively produced in adipose tissue, was analyzed three weeks after induction (Figure 5). Oil Red O staining was used to visualise lipid droplets within the cultures, this being the phenotypic signature of fat cells. Under control conditions, PPARγ2 is expressed in all sub-populations at a similar level (about 1 x 10^-3^ relative to GAPDH), and adiponectin expression is very low, i.e. almost at the detection limit of the RT-PCR (Figure 5). After adipogenic induction, PPARγ2 was slightly, but significantly decreased by a factor of 5 (p < 0.05). Only minor differences could be seen between the sub-populations. At the same time, adiponectin expression was induced for most of the cultures about 100- to 500-fold. This difference in comparison to the control cultures was significant. Cho5 culture and disc cell culture no. 2 showed alternating behaviour and were not induced in all cases. Under induction, W5C5+ showed lower adiponectin expression than W5C5-, and W8B2- lower than W8B2+; however, the effects were not significant. No significant differences could be detected between the subpopulations, neither under control nor under induction conditions. The rise in adiponectin mRNA expression did not correlate with the Oil Red O staining. Only very rare, sporadically positive cells could be found under induced conditions (Figure 5). In none of the control cultures were lipid droplets found. No obvious differences could be detected between disc cells and chondrocytes expanded in MSC growth medium as compared to standard chondrocyte cultures. Again, cultures in MSC growth medium were propagated to exclude negative selection of putative present stem cells by the medium. (For further information, see Additional file 1: Table S5.)

### Osteogenic differentiation

To determine the osteogenic differentiation potential of chondrocytic sub-populations gene expression of the transcription factor CBFA1 (RUNX2) and alkaline phosphatase were analyzed three weeks after induction (Figure 6). On the protein level, ALP activity was measured and mineralization of the cultures was detected by Alizarin Red staining. At first, the gene expression data of the osteogenic marker genes appeared to be more heterogeneous than their adipogenic counterparts. CBFA expression was between 7 x 10^-6^ and 2 x10^-4^ and ALP expression between 7 x 10^-7^ and 1 x10^-6^ (neglecting the outlier disc3 W5C5-). At the endpoint of the experiment, CBFA1 and ALP expression of control cultures was significantly different to cultures under induction conditions. ANOVA analysis revealed no differences between the individual sub-populations. Some cultures responded better to the stimulation whilst others did not. Interestingly, for some cultures, it appeared that osteogenic induction and adipogenic induction behave inversely. Good responders to osteogenic induction were bad responders to adipogenic induction (e.g. Disc2), good responders to adipogenic differentiation did not respond well to osteogenic induction (e.g. Cho8 MSCGM). The two W8B2+ sub-populations and the disc2 culture showed the highest stimulation index, with the ALP mRNA expression have increased more than 100-fold after stimulation. The disc2 culture also showed the highest ALP activity of all cultures (Figure 6 bottom, left side). The increase in ALP activity was about 3000 pmol/min/μg DNA after induction. An increase of about 2000 pmol/min/μg DNA could be detected for Cho4. The other cultures responded only to a minor degree to the stimulation and showed almost negligible or only a very moderate increase in ALP activity. However, the mean of the controls (285.0 pmol/min/μg DNA, n = 25) differed significantly (p < 0.05) from the mean of induced samples (1370.7 pmol/min/μg DNA, n = 25). Differences between sub-populations were not detected. High ALP activity levels did not inevitably lead to mineralization (at least at the time-point of the analysis). Disc2 as well as Cho4 cultures were negative in the Alizarin Red staining, showing no signs of mineralization (Figure 6 bottom, right). Only two cultures were positive for Alizarin Red: both cultures were expanded in MSC growth medium (Cho7 MSCGM, Cho8 MSCGM). But both showed only low ALP activity at that time-point (three weeks after induction). The corresponding culture of Cho8, which was expanded in chondrocyte culture medium, was Alizarin Red negative. Not a single culture was positive in the Alizarin Red staining under control conditions, which demonstrates that no spontaneous osteogenic differentiation occurred. (For further details, see Additional file 1: Table S6.)

### Chondrogenic differentiation

To determine the chondrogenic differentiation potential of chondrocytic sub-populations, a hydrogel system was used. This hydrogel is already used in a clinical study for disc regeneration (http://www.clinicaltrials.gov; identifier:NCT01640457) and we recently described chondrogenic differentiation of human MSCs in this hydrogel [34]. Four weeks after chondrogenic induction, the gene expression of chondrogenic marker genes collagen type II (COL2), and the transcription factors SOX9 were determined and compared to the level of monolayer (ML) cells before being embedded into the hydrogels (Figure 7). On the protein level, the GAG synthesis was analyzed in the hydrogels and the cumulated culture supernatants. The expression rate of SOX9 was the highest in comparison to the other transcription factors, CBFA1 and PPARγ2, and was expressed very uniformly in all cultures at an average value of 4 x 10^-3^ relative to GAPDH in monolayers, and of 3 x 10^-2^ in hydrogels (Figure 7 top). Under both hydrogel conditions, cells showed a significantly higher SOX9 expression as compared to the ML cells. In induced cells, this value decreased slightly to an average of 2 x 10^-2^ compared to cells in control gels, but there was no significant difference between the two gel groups. Under the same experimental conditions, no differences were found between the sub-populations.

The conversion of the two-dimensional monolayer into the three-dimensional environment in the hydrogel was sufficient to increase the collagen type II expression significantly (p < 0.05). Compared to the expression values at the end of the expansion, cells in hydrogels showed a higher collagen type II expression. In particular, both disc cell cultures re-differentiated very well in the hydrogel, showing a more than 1000-fold higher expression compared to the state before hydrogel culture. The addition of TGFβ_3_ to these hydrogel cultures did not improve collagen type II expression further. Collagen type II mRNA levels were variable and no difference between control and induction conditions could be detected. Expression levels ranged between 7 x 10^-6^ and 1 x 10^-1^ in controls, and 1 x 10^-5^ and 6 x 10^-2^ in induced cultures (see Additional file 1: Table S7). Compared to collagen type II, aggrecan mRNA expression values were very constant (data not shown). The average expression of all control cultures (3.99 x 10^-2^) was almost the same as for the induced cultures (3.64 x 10^-2^). Although no changes between control gels and gels under induction conditions were detected in the expression of the chondrogenic marker genes, a significant increase in the expression of the reference gene GAPDH was measured (Figure 7 bottom). Quantification cycles (C_q_) were around four cycles lower, meaning that 16-fold more GAPDH mRNA was present in the cultures stimulated by TGFβ_3_ as compared to the control cultures. This result was in accordance with an observation made regarding the viability of these cells. For the disc3 culture, a live-dead-staining was performed at the end of the chondrogenic differentiation. It defined the viability of control cells at about 36% (i.e., the majority of cells failed to survive the 3D-culture conditions) whereas the viability in the TGFβ_3_-stimulated cultures was about 80% (data not shown). To control this finding we analyzed the amount of RNA obtained after isolation. We found significantly (p < 0.05) lower RNA concentrations in the controls (mean: 10.6 μg/ml) compared to the induced samples (mean: 22.5 μg/ml). This result confirms the viability data.

While aggrecan mRNA is constantly expressed in control and induced cultures, a significant increase in GAG release was measured in the induced populations (p < 0.05). The cumulated GAG content doubled in the induced populations. A comparison of the sub-populations also showed no significant differences. An overview of the biochemical analyses is presented in Table 3.

**Table 3:** Biochemical analysis of hydrogel cultures after chondrogenic induction

**Sample**	**DNA hydrogel**		**GAG hydrogel/DNA**		**GAG supernatant/DNA**		**GAG/DNA**	
	**[μg]**		**[μg/μg]**		**[μg/μg]**		**[μg/μg]**	
	**Mean**	**sd**	**Mean**	**sd**	**Mean**	**sd**	**Mean**	**sd**
**control**								
Ø	2.04	0.86	0.86	0.38	5.46	3.06	6.35	2.86
W5C5+	2.55	0.78	0.83	0.21	3.27	2.54	4.10	2.65
W5C5-	2.16	0.68	1.03	0.14	5.23	4.05	6.25	4.06
W8B2+	2.66	0.09	0.99	0.05	6.10	0.40	7.10	0.45
W8B2-	2.04	0.45	0.97	0.17	11.60	5.02	12.57	5.16
**induction**								
Ø	2.42	1.08	1.79	0.49	10.48	4.26	12.27	4.63
W5C5+	2.63	1.23	1.71	0.72	8.90	4.86	10.61	5.40
W5C5-	2.35	1.25	1.82	0.87	10.63	5.43	12.45	6.16
W8B2+	3.27	0.70	1.79	0.57	9.36	2.00	11.15	2.57
W8B2-	3.36	0.26	1.68	0.39	8.42	1.37	10.10	1.75

The GAG synthesis rate per week ranged at about 1.8 μg GAG per μg DNA under control conditions. This was higher than the amount produced by cells being embedded into the hydrogel directly after MACS (about 1 μg / μg DNA, see Figure 1). After induction, the synthesis was further increased to about 2.8 μg GAG / μg DNA. Thus, even after prolonged expansion, under appropriate conditions, chondrocytes and disc cells were able to synthesize GAGs in a manner similar to cells with distinctly lower population doublings. Chondrocytes cultured in MSC growth medium produced less GAG/DNA as compared to chondrocytes cultured in chondrocyte medium. No difference was recorded between chondrocytes and disc cells. (For additional details, see Additional file 1: Tables S7 and S8).

### Expression of cell surface antigens and differentiation capacity as a function of population doublings *in vitro*

As only minor differences between the sub-populations were found after prolonged expansion, we additionally analyzed the adipogenic and osteogenic differentiation of chondrocytes directly after MACS (chondrogenic differentiation capacity was already indirectly compared; see Figures 1 and 7). Moreover, the surface antigen expression was analyzed at these two time-points. Directly after MACS, the W5C5+ and the W8B2+ fraction contained more than 97% positive cells, as shown by flow cytometry (Figure 8A, top row). In addition, there was no change in the proportions of distribution for W5C5+ in W8B2-sorted cells and vice versa (data not shown). At the end of expansion in chondrocyte medium, both the W5C5+ and the W8B2+ sub-populations lost a substantial amount of antigen-positive cells or lost the expression of the corresponding cell surface antigens (Figure 8A, bottom row). Finally, after more than 8 pds *in vitro*, the expression profiles for all three populations were more or less identical. This loss of antigen expression was not found for other MSC antigens, including CD49e, CD73, CD90, CD105, and CD166. Their expression remained stable during this time-span (data not shown). Also, differentiation capacity changed depending on the cumulative population doublings of the cultures. Under control conditions, the expression of adiponectin and alkaline phosphatase did not differ significantly (p > 0.05) dependent on the pds of the cells (Figure 8B,C). The ALP activity was also constant. In both groups (the “after MACS” group = “younger” and the “after MACS + > 8 pds” group = “older”), induction led to a statistically significant increase in adiponectin and ALP gene expression, and ALP activity (p < 0.05). (For reasons of clarity this was not plotted into the graphs.) “Younger” and “older” cells did not differ in their response to adipogenic induction. However, the osteogenic induction was much more pronounced in those cultures, which were differentiated directly after MACS. The average increase in ALP gene expression after induction was about 9-fold higher in all “younger” cell populations compared to “older” ones. The average ALP activity increased by a factor of 11 in “younger”, and increased only by a factor of 4 in the expanded “older” cultures. This effect was statistically significant for unsorted and W5C5+ populations on ALP gene level and for all three sub-populations on ALP activity level (Figure 8C). A comparison of the individual sub-populations under the same experimental conditions showed that there were no differences under control conditions. After osteogenic induction, the W5C5+ fraction differed significantly from the unsorted and the W5C5- fraction with regard to ALP activity. Alizarin Red staining indicating mineralization of the cultures was not consistent in the cultures (as seen for the differentiation after > 8 pds). One of three cultures was positive; two were negative (data not shown). In the adipogenic induction medium, both “*younger*” and “*older*” cells showed a significant increase in adiponectin mRNA; however, the expression remained at a very low level. In contrast to the osteogenic induction, the “*younger*” cells were more inducible directly after MACS; whereas the expanded *“older”* cells displayed a stronger response (this difference between the two groups of cells after adipogenic induction was not significant). In none of the cultures could lipid droplets be detected by means of Oil red O staining (data not shown), a finding that demonstrates that genotypic alterations do not necessarily manifest themselves as phenotypic changes. To summarize the results of these differentiation experiments, it can be stated that the W5C5+ cell populations, besides being highly chondrogenic, tend to a greater extent toward the osteogenic phenotype, rather than to an adipogenic one, once provoked into an alternative phenotype.

## Discussion

The presented experimental study contains four major results: Firstly, our results confirm and have reproduced other observations relating to MSC-like cells in articular cartilage. Secondly, these populations persist during propagation *in vitro*. Thirdly, de-differentiated populations retain chondrogenic properties above and beyond the accepted population doubling limits that now prevail (less than 6 pds). Fourthly, the observed osteogenic and adipogenic potency was chiefly observable at the level of gene expression and much less so at the level of cell function.

Taken together, the data indicate the presence of certain plasticity within chondrocytic cell populations which may be related to the presence of classical MSC sub-types but more likely to the presence of restricted precursor populations for skeletal phenotypes.

The motivation for our study was based on recent insights relating to the presence of MSCs or MSC-like cells in cartilage [4,8] and intervertebral disc tissue [2,3] and the perspectives and/or problems which possibly arise from such cells within the context of ACI. We investigated whether these cells in cartilage share true “stem cell” features and whether these “stem cell” features or the “stemness” of chondrocyte populations are advantageous or disadvantageous to ACI.

To test the MSC sub-population hypothesis, we carried out the following experiments: i) analysis of the presence of surface antigens described for MSCs, ii) generation of sub-populations which are enriched or depleted of cells presenting MSC surface antigens, iii) analysis of the differentiation state after separation, description of the replicative capacity, and differentiation potential into adipogenically, osteogenically, and chondrogenically functional cell types.

According to the position paper of the Society for Cellular Therapy, in which the minimal criteria for MSCs are stipulated, MSCs must adhere to tissue culture plastic when maintained in standard culture medium and have to show multi-potent differentiation potential. Furthermore, they must express CD73, CD90, and CD105, and must lack expression of CD34, CD45, CD14, CD79α, and HLA-DR surface molecules [41]. Two criteria, the adherence and expression of the mentioned CDs, are fulfilled by chondrocytes and also by IVD cells. Similar to MSCs, these cell types were 100% positive in our flow cytometry analysis (within the error brackets for the method) for the above-mentioned markers CD73, CD90, and CD105, as well as for other markers described in the literature as having been expressed on MSCs, such as CD49e, CD140b, and CD166. In contrast, CD271 was not expressed on cultured chondrocytes and disc cells. Similar flow cytometry profiles for putative MSCs isolated from degenerated nucleus pulposus were shown in a publication from Blanco and colleagues [6]. The expression of CD73, CD90, CD105, and CD166 on human chondrocytes has been also demonstrated by others [40]. More recently, W5C5 and W8B2 antigens have been added to the list of markers for chondrogenic and osteogenic MSCs from bone marrow [30-33].

We were able to establish W5C5 (SUSD2) and W8B2 (membrane alkaline phosphatase) sub-populations using MACS technology. Unfortunately, W8B2 sorting is not very practicable for most experimental set-ups due to the low amount of positive cells obtained after sorting. In general, MACS separations led to enriched fractions with a satisfactory rate of positive cells, as confirmed by flow cytometry of the MACS cells (on average between 80 and 90%). However, the depleted fractions still contained relatively high numbers of positive cells (on average about 30 to 40%). The impurity of the depleted MACS fractions might be one reason for the lack of difference between + and - fractions in our study. Cells showed good viability and the potential for chondrogenic maturation was not altered in comparison to the unsorted populations (Figure 1).

To test the stability of the sub-populations generated with respect to “stemness”, we expanded the cells after MACS for several passages until the cells had accumulated at least additional 8 pds. To exclude an undesired selection of cells by using a medium that was especially designed for chondrocytes (chondrocyte medium, CM), we expanded two cultures in a commercial MSC growth medium (MSCGM containing fibroblast growth factor-2 (FGF-2)). The result met our expectations: the cells became less differentiated and more proliferative. In addition, under all culture conditions, the individual cell populations tended to become more similar with increasing pds.

With regard to their differentiation potency, it can briefly be stated that, under control conditions, chondrocytes and chondrocyte sub-populations were able to express the master transcription factors for all three tested lineages: PPARγ2, CBFA1, and SOX9 and lineage-related specific genes such as adiponectin, alkaline phosphatase, and collagen type II. This might be a primordial pattern for all cells of mesodermal origin, since, during embryogenesis, the fate of the cells is defined by environmental cues and not by lineage-specific origin [42-44]. Transcription factors seem to be expressed constitutively and stably in all cultures and under all conditions. However, the mRNA expression for tissue-specific genes did not necessarily lead to a detectable functional cell/tissue phenotype. Under control conditions, neither the formation of lipid droplets nor mineralization was detected. On the other hand, and unlike adipogenic and osteogenic differentiation, chondrogenic differentiation could be induced simply by transferring the cells from monolayer to a three-dimensional culture in our hydrogel, without the use of induction medium (concerning viability of cultures, see discussion below). Good chondrogenic differentiation after several passages and pds showed slight discrepancy to some published data. Giovannini *et al.* determined cumulative pds of human articular chondrocytes expanded in monolayer culture and identified a threshold range of 3.57 and 4.19 pds as indicative of a loss of intrinsic chondrogenic capacity in pellets [23]. Kang *et al.* showed an effect of passage number on tissue-engineered cartilage when cells of different passages were cultured on biodegradable polymer scaffolds. Passage 5 chondrocytes did not produce cartilage-specific extracellular matrices [22]. Peltari showed the loss of the ectopic cartilage formation capacity of human chondrocytes *in vivo* to be between 0 and 6 pds [24].

These differences could possibly be caused by different culture conditions rather than by the intrinsic failure of chondrocyte differentiation pathways. Recently, Schuh *et al.* demonstrated that the availability of adhesion sites within agarose hydrogels inhibited the cellular re-differentiation [45]. The hydrogel used in this study is based on human serum albumin that does not contain adhesive peptide residues for chondrocytes [46].

After adipogenic induction, expression of adiponectin gene expression was up-regulated in most cultures by a factor of 100 – 500. But only very sporadically were Oil Red O positive cells with lipid droplets found.

After osteogenic differentiation, an increase in ALP mRNA transcripts and enzyme activity were found for some cultures, but non-responders were also found. One explanation for the inconsistent results may be rooted in the mineralization process, which may be related to the induction of osteoblasts or hypertrophic chondrocytes from these cells with low differentiation levels. ALP mRNA expression and ALP activity do not correlate with mineralization. Interestingly, we found signs of mineralization only in those two cultures that were expanded in a commercial MSC growth medium. This might be due to the presence of FGF-2 during the expansion period. In general, the expression of ALP and osteogenic differentiation in the context of chondrocytes is an unresolved issue. In skeletal development, cartilaginous anlagen are remodeled to bone. Thus the fate of most chondrocytes is to develop into mineralizing cells. Therefore, the ability to express osteogenic marker molecules must be an intrinsic property of the chondrocyte. ALP may have multiple functions in skeletal tissue and its expression is not causally linked to chondrocyte hypertrophy [47]. Indications for such processes are also described for osteoarthritic cartilage and in degenerative discs [48].

To further analyze this problem, we compared mRNA expression of osteocalcin and bone sialoprotein-2 (BSP-2) in mineralizing sub-populations and in highly ALP-positive but non-mineralizing sub-populations. Both pools generated the same level of osteocalcin mRNA, whereas the non-mineralizing cells expressed even more BSP-2 (about 100-fold, data not shown). This result suggests that other regulatory mechanisms, such as miRNA expression, may be involved in the process of mineralization [49-52]. In addition, the expression of ALP mRNA was still about 100-fold lower in chondrocytes cultures as compared to the respective mRNA levels of three commercially-available MSC cultures (data not shown). Taken together, cell populations obtained from cartilage/disc tissue seem to have a lower tendency to mineralize than typical bone marrow MSCs.

This corrobates with clinical results of studies on the repair of cartilage defects using microfracture or ACI, respectively. In the case of microfracture, the intra-lesional formation of osteophytes is frequently described, but this has been never reported for ACI [53-56]. Microfracture is a procedure in which, by breaking through the subchondral plate, bone marrow cells (potentially containing multipotent MSCs) are able to migrate into the defective area of a joint cartilage trauma and rebuild repair cartilage, but may also cause the undesired formation of osteophytes. In contrast, ACI uses *in vitro* expanded chondrocytes isolated from autologous cartilage and may therefore be less prone to undesired mineralization.

The results from chondrogenic induction demonstrated that chondrocytes and disc cells were able to synthesize major cartilage extracellular matrix components on the mRNA and protein level, even after prolonged expansion. Interestingly, we found a relatively high number of dead cells in the hydrogel under control conditions, but not after stimulation with TGF-β_3_. This is not the case when cells expanded in primary culture where embedded into the hydrogel [34]. The transfer into hydrogel may generate selection pressure on the cells, leading to the elimination of non-adaptive cells from the culture. An important implication of such a finding is that a three-dimensional environment may actually suppress untoward adaptations or differentiations. TGF-β supplementation improves the viability of the cultures and leads to an increase in GAG synthesis. This positive influence on survival and function has been reported by Risbud *et al.* in terms of intervertebral disc (IVD) cells in organ cultures [57].

With regard to the presence of MSCs or other precursor cell types in articular cartilage and intervertebral discs, the literature presents a rather complex picture (Table 4). In a study comparable to ours, Pretzel *et al.* used CD105/CD166 as markers [29]. Differentiation into all three lineages were described and clear differences between the + and - fractions were only seen in the chondrogenic differentiation. Unfortunately, this study lacks an unsorted chondrocyte population as a baseline control. Blanco and colleagues isolated and characterized cells from human degenerated nucleus pulposus and compared their characteristics to MSCs obtained from the bone marrow of the same patients [6]. They showed that the immunophenotypic profiles of both cell types were similar and that chondrogenic genes were expressed. But, as in the study by Pretzel, a comparison with unsorted nucleus pulposus (NP) cells is missing. The authors concluded that those cells were MSCs despite the absence of adipogenic differentiation. Risbud and colleagues also reported only a small percentage of cells which displayed evidence of adipogenic differentiation in skeletal progenitor cells from IDV [3]. Liu *et al.* described cells derived from the degenerated human IVD endplate and demonstrated a differentiation capacity comparable to that of bone marrow MSCs [2].

**Table 4:** Overview of selected literature

**Ref. no.**	**Cell source**	**Sorting**	**Selective culture**	**Clonality**	**Potency**		
					**Adipogenic**	**Osteogenic**	**Chondrogenic**
[2]	degenerated IVD endplate	no	agarose	no	+/-	+	+
[3]	degenerated AF and NP	CD 133 +/-	no	no	+/-	+/-	+
[6]	degenerated NP	no	no	no	-	+	+
[8]	articular cartilage	no	agarose	yes (cluster)	+/-	+/-	+/-
	(ACI surgery)						
[26]	normal articular cartilage	no	TGFb1, FGF-2, PDGF-BB	yes (n = 20 clones)	20% of clones	25% of clones	60% of clones
				2 A/O/C			
[26]	normal articular cartilage	no	no	yes (n = 7 clones)	57% of clones	28% of clones	57% of clones
				1 A/O/C			
[28]	articular cartilage (knee surgery)	no	fibronectin coating, osteogenic differentiation in pellet culture	yes	+/-	+	+
[29]	OA cartilage	CD166 +/-	no	no	+/-	+	+
[58]	articular cartilage	no	no	yes (n = 53)	+/-	+	+
	(ACI surgery)			17 A/O/C			
[59]	OA cartilage	no	no		not done	-	+
[60]	normal, OA cartilage	CD 155+/CD166+	no	no	+/-	+	+
[61]	normal, OA cartilage	Hoechst dye 33342	no	no tissue immunostaining	-	+/-	+
presented study	normal, OA cartilage, IVD	W5C5 +/-W8B2 +/-	no	no	+/-	+/-	+

+/- = differentiation either only by gene expression or only spotty distribution in Oil Red staining or mineral deposition or chondrogenesis.

None of the studies mentioned above, including our own, performed clonal experiments. Therefore it is possible that a multi-lineage differentiation potential may be related to a pre-existing mixture of differentially committed progenitor cells. To demonstrate the presence of multi-potent cells in a cell population, it would be essential to use clonal cell populations. Barbero *et al.* demonstrated that multi-clonal populations of human articular chondrocytes exhibit differentiation plasticity. This plasticity could be modulated by growth factors used during expansion. Plasticity was highly heterogeneous across different clones [26]. Only two out of 20 clones differentiated toward the adipogenic, osteogenic, and chondrogenic lineage. Most of the expanded clones were able to re-differentiate only into chondrocytes or were unable to differentiate. De la Fuente reported that 17 out of 53 clones were able to differentiate into the three lineages [58]. At a clonal level, Williams and colleagues identified a cartilage progenitor cell, resident in human articular cartilage [28]. They showed that the progenitor cells have the capacity to differentiate into the adipogenic lineage, whilst retaining a restricted differential potential during osteogenic differentiation when investigated in a 3D pellet culture system. However, as in our study, unselected chondrocytes were also positive for differentiation into the three lineages. However, the overall efficacy of differentiation seems to be very low in comparison to MSCs isolated from bone marrow.

Our study revealed unexpectedly few differences between the sub-populations and the unselected chondrocyte or disc cell cultures. During re-testing in the presence of surface antigens, we observed a reversal in the expression of the markers in the positive fraction, for both W5C5+ and W8B2+ cells, to a level defined by the unsorted initial cells. This tendency was described also by Pretzel *et al.* for CD166+ cells [29]. Interestingly, other markers were expressed until expansion ended, with no change in proportions. To verify that we did not have a bias against cells with stem cell characteristics during expansion, we repeated adipogenic and osteogenic differentiation experiments directly after MACS of the expanded cells (PO, “younger”): we found that the older cultures seemed to lose their osteogenic differentiation capacity, but gained adipogenic potential, as evidenced by the levels of the explored functional tissue markers. The stability of such findings is underscored by a recent work including an inter-laboratory comparison on MSC differentiation, with the result that chondrogenesis may even serve as a means of regulatory demanded quality control measures [62]. As our culture conditions apparently preserved chondrogenic features and suppressed osteogenic or adipogenic lineages, *in vitro* expansion of chondrocytes in ACI may actually be an advantage rather than a disadvantage for the formation of sound repair cartilage.

## Conclusion

The expression of putative MSC marker genes on chondrocytes and disc cells does not necessarily determine the plasticity of cells. W5C5 and W8B2 antigen expression might be of prognostic value to MSCs, but is not predictive in terms of cartilage and disc tissue-derived chondrocytes. Expression of MSC markers is, at best, an inclusive feature to characterize multi-potent stem cells, but not an exclusive criterion for chondrogenetic properties. Our results demonstrate that, under established *in vitro* culture conditions, a chondroid precursor, but not a multi-potent mesenchymal, cell type can be obtained. We demonstrate that, at a very low level, a certain kind of multi-differentiation potential is present in the chondrocyte population after stimulation, but the affinity to the chondrogenic lineage is strongly pronounced. As a consequence, even after forced enrichment of putative MSC-like cell populations, *in vitro* expanded chondrocyte cultures do not pose a major risk of adverse adipose or osseous differentiation, even when such differentiation was induced *in vitro*. Appropriate cell culture conditions during expansion (medium especially designed for chondrocytes) and differentiation (hydrogel adapted to the need of chondrocytes/disc cells) favor the development of a chondrogenic phenotype – as proposed for ACI. However, using a stem cell medium, it is possible to generate a more osteogenic phenotype artificially. In summary, it can be stated that the “stemness” of chondrocytes expanded for use in ACI is not a potential risk, and represents an advantage instead, when cells are handled in a proper manner.

## Competing interests

KB and JAM have applied for a patent relating to the application of the hydrogel material.

## Authors’ contributions

KB contributed to the conception and design of the study, MACS experiments, cell culture and differentiation experiments, biochemical and histological analysis, interpretation of data, statistical analysis, writing the manuscript. CS carried out flow cytometry. CF carried out gene expression analysis. JAM helped conceive the study, participated in discussions and interpretation of data, and in the drafting and revision of the manuscript. WKA took over supervision, discussion and interpretation of data, and revision of the manuscript. All authors read and approved the final manuscript.

## Supplementary Material

Additional file 1: Table S1 Human tissues used for MACS / cell culture experiments. **Table S2.** Antibodies used for flow cytometry. **Table S3.** Primers used for gene expression analysis. **Table S4.** Gene expression of chondrocytic subpopulations dependent on the population doublings. **Table S5.** Adipogenic differentiation: relative gene expression data. **Table S6.** Osteogenic differentiation: relative gene expression data. **Table S7.** Chondrogenic differentiation: relative gene expression data. **Table S8.** Chondrogenic differentiation: GAG synthesis. Click here for file

Additional file 2: Figure S1 Experimental set-up for MACS sorting experiments. **Figure S2.** Experimental set-up to characterise W5C5 and W8B2 positive subpopulations regarding MSC characteristics. Click here for file

## References

[B1] Pittenger MF Mackay AM Beck SC Jaiswal RK Douglas R Mosca JD Moorman MA Simonetti DW Craig S Marshak DR Multilineage potential of adult human mesenchymal stem cells Science 1999 284 143 147 10.1126/science.284.5411.143 10102814

[B2] Liu LT Huang B Li CQ Zhuang Y Wang J Zhou Y Characteristics of stem cells derived from the degenerated human intervertebral disc cartilage endplate PLoS One 2011 6 e26285 10.1371/journal.pone.0026285 22028847 PMC3196539

[B3] Risbud MV Guttapalli A Tsai TT Lee JY Danielson KG Vaccaro AR Albert TJ Gazit Z Gazit D Shapiro IM Evidence for skeletal progenitor cells in the degenerate human intervertebral disc Spine (Phila Pa 1976) 2007 32 2537 2544 10.1097/BRS.0b013e318158dea6 17978651

[B4] Hattori S Oxford C Reddi AH Identification of superficial zone articular chondrocyte stem/progenitor cells Biochem Biophys Res Commun 2007 358 99 103 10.1016/j.bbrc.2007.04.142 17482567 PMC2583246

[B5] Koelling S Kruegel J Irmer M Path JR Sadowski B Miro X Miosge N Migratory chondrogenic progenitor cells from repair tissue during the later stages of human osteoarthritis Cell Stem Cell 2009 4 324 335 10.1016/j.stem.2009.01.015 19341622

[B6] Blanco JF Graciani IF Sanchez-Guijo FM Muntion S Hernandez-Campo P Santamaria C Carrancio S Barbado MV Cruz G Gutierrez-Cosio S Isolation and characterization of mesenchymal stromal cells from human degenerated nucleus pulposus: comparison with bone marrow mesenchymal stromal cells from the same subjects Spine (Phila Pa 1976) 2010 35 2259 2265 10.1097/BRS.0b013e3181cb8828 20622750

[B7] Tallheden T Brittberg M Peterson L Lindahl A Human articular chondrocytes–plasticity and differentiation potential Cells Tissues Organs 2006 184 55 67 10.1159/000098947 17361078

[B8] Thornemo M Tallheden T Sjogren Jansson E Larsson A Lovstedt K Nannmark U Brittberg M Lindahl A Clonal populations of chondrocytes with progenitor properties identified within human articular cartilage Cells Tissues Organs 2005 180 141 150 10.1159/000088242 16260860

[B9] da Silva ML Caplan AI Nardi NB In search of the in vivo identity of mesenchymal stem cells Stem Cells 2008 26 2287 2299 10.1634/stemcells.2007-1122 18566331

[B10] da Silva ML Chagastelles PC Nardi NB Mesenchymal stem cells reside in virtually all post-natal organs and tissues J Cell Sci 2006 119 2204 2213 10.1242/jcs.02932 16684817

[B11] Shi S Gronthos S Perivascular niche of postnatal mesenchymal stem cells in human bone marrow and dental pulp J Bone Miner Res 2003 18 696 704 10.1359/jbmr.2003.18.4.696 12674330

[B12] Zannettino AC Paton S Arthur A Khor F Itescu S Gimble JM Gronthos S Multipotential human adipose-derived stromal stem cells exhibit a perivascular phenotype in vitro and in vivo J Cell Physiol 2008 214 413 421 10.1002/jcp.21210 17654479

[B13] Feng J Mantesso A Sharpe PT Perivascular cells as mesenchymal stem cells Expert Opin Biol Ther 2010 10 1441 1451 10.1517/14712598.2010.517191 20836622

[B14] Crisan M Yap S Casteilla L Chen CW Corselli M Park TS Andriolo G Sun B Zheng B Zhang L A perivascular origin for mesenchymal stem cells in multiple human organs Cell Stem Cell 2008 3 301 313 10.1016/j.stem.2008.07.003 18786417

[B15] Paul G Ozen I Christophersen NS Reinbothe T Bengzon J Visse E Jansson K Dannaeus K Henriques-Oliveira C Roybon L The adult human brain harbors multipotent perivascular mesenchymal stem cells PLoS One 2012 7 e35577 10.1371/journal.pone.0035577 22523602 PMC3327668

[B16] Brittberg M Lindahl A Nilsson A Ohlsson C Isaksson O Peterson L Treatment of deep cartilage defects in the knee with autologous chondrocyte transplantation N Engl J Med 1994 331 889 895 10.1056/NEJM199410063311401 8078550

[B17] Meisel HJ Siodla V Ganey T Minkus Y Hutton WC Alasevic OJ Clinical experience in cell-based therapeutics: disc chondrocyte transplantation A treatment for degenerated or damaged intervertebral disc Biomol Eng 2007 24 5 21 10.1016/j.bioeng.2006.07.002 16963315

[B18] Mayne R Vail MS Mayne PM Miller EJ Changes in type of collagen synthesized as clones of chick chondrocytes grow and eventually lose division capacity Proc Natl Acad Sci U S A 1976 73 1674 1678 10.1073/pnas.73.5.1674 1064040 PMC430362

[B19] Benya PD Padilla SR Nimni ME Independent regulation of collagen types by chondrocytes during the loss of differentiated function in culture Cell 1978 15 1313 1321 10.1016/0092-8674(78)90056-9 729001

[B20] Darling EM Athanasiou KA Rapid phenotypic changes in passaged articular chondrocyte subpopulations J Orthop Res 2005 23 425 432 10.1016/j.orthres.2004.08.008 15734258

[B21] Benya PD Shaffer JD Dedifferentiated chondrocytes reexpress the differentiated collagen phenotype when cultured in agarose gels Cell 1982 30 215 224 10.1016/0092-8674(82)90027-7 7127471

[B22] Kang SW Yoo SP Kim BS Effect of chondrocyte passage number on histological aspects of tissue-engineered cartilage Biomed Mater Eng 2007 17 269 276 17851169

[B23] Giovannini S Diaz-Romero J Aigner T Mainil-Varlet P Nesic D Population doublings and percentage of S100-positive cells as predictors of in vitro chondrogenicity of expanded human articular chondrocytes J Cell Physiol 2010 222 411 420 10.1002/jcp.21965 19890919

[B24] Pelttari K Lorenz H Boeuf S Templin MF Bischel O Goetzke K Hsu HY Steck E Richter W Secretion of matrix metalloproteinase 3 by expanded articular chondrocytes as a predictor of ectopic cartilage formation capacity in vivo Arthritis Rheum 2008 58 467 474 10.1002/art.23302 18240244

[B25] Diaz-Romero J Gaillard JP Grogan SP Nesic D Trub T Mainil-Varlet P Immunophenotypic analysis of human articular chondrocytes: changes in surface markers associated with cell expansion in monolayer culture J Cell Physiol 2005 202 731 742 10.1002/jcp.20164 15389573

[B26] Barbero A Ploegert S Heberer M Martin I Plasticity of clonal populations of dedifferentiated adult human articular chondrocytes Arthritis Rheum 2003 48 1315 1325 10.1002/art.10950 12746904

[B27] Tallheden T Dennis JE Lennon DP Sjogren-Jansson E Caplan AI Lindahl A Phenotypic plasticity of human articular chondrocytes J Bone Joint Surg Am 2003 85-A Suppl 2 93 100 12721350 10.2106/00004623-200300002-00012

[B28] Williams R Khan IM Richardson K Nelson L McCarthy HE Analbelsi T Singhrao SK Dowthwaite GP Jones RE Baird DM Identification and clonal characterisation of a progenitor cell sub-population in normal human articular cartilage PLoS One 2010 5 e13246 10.1371/journal.pone.0013246 20976230 PMC2954799

[B29] Pretzel D Linss S Rochler S Endres M Kaps C Alsalameh S Kinne RW Relative percentage and zonal distribution of mesenchymal progenitor cells in human osteoarthritic and normal cartilage Arthritis Res Ther 2011 13 R64 10.1186/ar3320 21496249 PMC3132059

[B30] Battula VL Treml S Bareiss PM Gieseke F Roelofs H de Zwart P Muller I Schewe B Skutella T Fibbe WE Isolation of functionally distinct mesenchymal stem cell subsets using antibodies against CD56, CD271, and mesenchymal stem cell antigen-1 Haematologica 2009 94 173 184 10.3324/haematol.13740 19066333 PMC2635396

[B31] Alexander D Schafer F Olbrich M Friedrich B Buhring HJ Hoffmann J Reinert S MSCA-1/TNAP selection of human jaw periosteal cells improves their mineralization capacity Cell Physiol Biochem 2010 26 1073 1080 10.1159/000323985 21220938

[B32] Buhring HJ Battula VL Treml S Schewe B Kanz L Vogel W Novel markers for the prospective isolation of human MSC Ann N Y Acad Sci 2007 1106 262 271 10.1196/annals.1392.000 17395729

[B33] Masuda H Anwar SS Buhring HJ Rao JR Gargett CE A novel marker of human endometrial mesenchymal stem-like cells Cell Transplant 2012 21 2201 2214 10.3727/096368911X637362 22469435

[B34] Benz K Freudigmann C Mueller J Wurst H Albrecht D Badke A Gaissmaier C Mollenhauer J A polyethylene glycol-crosslinked serum albumin/hyaluronan hydrogel for the cultivation of chondrogenic cell types Adv Eng Mater 2010 12 B539 551 10.1002/adem.201080028

[B35] Benz K Stippich C Osswald C Gaissmaier C Lembert N Badke A Steck E Aicher WK Mollenhauer JA Rheological and biological properties of a hydrogel support for cells intended for intervertebral disc repair BMC Musculoskelet Disord 2012 13 54 10.1186/1471-2474-13-54 22490206 PMC3375205

[B36] Bustin SA Benes V Garson JA Hellemans J Huggett J Kubista M Mueller R Nolan T Pfaffl MW Shipley GL The MIQE guidelines: minimum information for publication of quantitative real-time PCR experiments Clin Chem 2009 55 611 622 10.1373/clinchem.2008.112797 19246619

[B37] Gebhard PM Gehrsitz A Bau B Soder S Eger W Aigner T Quantification of expression levels of cellular differentiation markers does not support a general shift in the cellular phenotype of osteoarthritic chondrocytes J Orthop Res 2003 21 96 101 10.1016/S0736-0266(02)00094-3 12507585

[B38] Altschul SF Madden TL Schaffer AA Zhang J Zhang Z Miller W Lipman DJ Gapped BLAST and PSI-BLAST: a new generation of protein database search programs Nucleic Acids Res 1997 25 3389 3402 10.1093/nar/25.17.3389 9254694 PMC146917

[B39] Chandrasekhar S Esterman MA Hoffman HA Microdetermination of proteoglycans and glycosaminoglycans in the presence of guanidine hydrochloride Anal Biochem 1987 161 103 108 10.1016/0003-2697(87)90658-0 3578776

[B40] Cournil-Henrionnet C Huselstein C Wang Y Galois L Mainard D Decot V Netter P Stoltz JF Muller S Gillet P Watrin-Pinzano A Phenotypic analysis of cell surface markers and gene expression of human mesenchymal stem cells and chondrocytes during monolayer expansion Biorheology 2008 45 513 526 18836250

[B41] Dominici M Le Blanc K Mueller I Slaper-Cortenbach I Marini F Krause D Deans R Keating A Prockop D Horwitz E Minimal criteria for defining multipotent mesenchymal stromal cells. The international society for cellular therapy position statement Cytotherapy 2006 8 315 317 10.1080/14653240600855905 16923606

[B42] Chou AI Reza AT Nicoll SB Distinct intervertebral disc cell populations adopt similar phenotypes in three-dimensional culture Tissue Eng Part A 2008 14 2079 2087 10.1089/ten.tea.2007.0337 18636941 PMC2809660

[B43] Lash J Holtzer S Holtzer H An experimental analysis of the development of the spinal column. VI. Aspects of cartilage induction Exp Cell Res 1957 13 292 303 10.1016/0014-4827(57)90008-3 13480296

[B44] Wolpert L Principles of Develpoment 1998 London, New York: Current Biology LTD

[B45] Schuh E Hofmann S Stok K Notbohm H Muller R Rotter N Chondrocyte redifferentiation in 3D: the effect of adhesion site density and substrate elasticity J Biomed Mater Res A 2012 100 38 47 21972220 10.1002/jbm.a.33226

[B46] Kuschel C Steuer H Maurer AN Kanzok B Stoop R Angres B Cell adhesion profiling using extracellular matrix protein microarrays Biotechniques 2006 40 523 531 10.2144/000112134 16629399

[B47] Miyake T Cameron AM Hall BK Variability of embryonic development among three inbred strains of mice Growth Dev Aging 1997 61 141 155 9546105

[B48] Rutges JP Duit RA Kummer JA Oner FC van Rijen MH Verbout AJ Castelein RM Dhert WJ Creemers LB Hypertrophic differentiation and calcification during intervertebral disc degeneration Osteoarthr Cartil 2010 18 1487 1495 10.1016/j.joca.2010.08.006 20723612

[B49] Zhang ZJ Zhang H Kang Y Sheng PY Ma YC Yang ZB Zhang ZQ Fu M He AS Liao WM miRNA expression profile during osteogenic differentiation of human adipose-derived stem cells J Cell Biochem 2012 113 888 898 10.1002/jcb.23418 22371969

[B50] Schaap-Oziemlak AM Raymakers RA Bergevoet SM Gilissen C Jansen BJ Adema GJ Kogler G le Sage C Agami R van der Reijden BA Jansen JH MicroRNA hsa-miR-135b regulates mineralization in osteogenic differentiation of human unrestricted somatic stem cells Stem Cells Dev 2010 19 877 885 10.1089/scd.2009.0112 19795981

[B51] Eskildsen T Taipaleenmaki H Stenvang J Abdallah BM Ditzel N Nossent AY Bak M Kauppinen S Kassem M MicroRNA-138 regulates osteogenic differentiation of human stromal (mesenchymal) stem cells in vivo Proc Natl Acad Sci U S A 2011 108 6139 6144 10.1073/pnas.1016758108 21444814 PMC3076836

[B52] Zhang Y Xie RL Croce CM Stein JL Lian JB van Wijnen AJ Stein GS A program of microRNAs controls osteogenic lineage progression by targeting transcription factor Runx2 Proc Natl Acad Sci U S A 2011 108 9863 9868 10.1073/pnas.1018493108 21628588 PMC3116419

[B53] Proffen B von Keudell A Vavken P Evidence-based therapy for cartilage lesions in the knee - regenerative treatment options Z Orthop Unfall 2012 150 280 289 22723070 10.1055/s-0031-1298387

[B54] Niemeyer P Koestler W Sudkamp NP Problems and complications of surgical techniques for treatment of full-thickness cartilage defects Z Orthop Unfall 2011 149 45 51 10.1055/s-0030-1250104 20648422

[B55] Dhollander AA Verdonk PC Lambrecht S Almqvist KF Elewaut D Verbruggen G Verdonk R The combination of microfracture and a cell-free polymer-based implant immersed with autologous serum for cartilage defect coverage Knee Surg Sports Traumatol Arthrosc 2011 20 1773 1780 22068269 10.1007/s00167-011-1763-y

[B56] Fortier LA Cole BJ McIlwraith CW Science and animal models of marrow stimulation for cartilage repair J Knee Surg 2012 25 3 8 22624241 10.1055/s-0032-1310389

[B57] Risbud MV Di Martino A Guttapalli A Seghatoleslami R Denaro V Vaccaro AR Albert TJ Shapiro IM Toward an optimum system for intervertebral disc organ culture: TGF-beta 3 enhances nucleus pulposus and anulus fibrosus survival and function through modulation of TGF-beta-R expression and ERK signaling Spine (Phila Pa 1976) 2006 31 884 890 10.1097/01.brs.0000209335.57767.b5 16622376

[B58] de la Fuente R Abad JL Garcia-Castro J Fernandez-Miguel G Petriz J Rubio D Vicario-Abejon C Guillen P Gonzalez MA Bernad A Dedifferentiated adult articular chondrocytes: a population of human multipotent primitive cells Exp Cell Res 2004 297 313 328 10.1016/j.yexcr.2004.02.026 15212937

[B59] Bernstein P Sperling I Corbeil D Hempel U Fickert S Progenitor cells from cartilage - no osteoarthritis-grade-specific differences in stem cell marker expression Biotechnol Prog 2012 Epub: 2012/11/23 10.1002/btpr.1668 23172745

[B60] Alsalameh S Amin R Gemba T Lotz M Identification of mesenchymal progenitor cells in normal and osteoarthritic human articular cartilage Arthritis Rheum 2004 50 1522 1532 10.1002/art.20269 15146422

[B61] Grogan SP Miyaki S Asahara H D’Lima DD Lotz MK Mesenchymal progenitor cell markers in human articular cartilage: normal distribution and changes in osteoarthritis Arthritis Res Ther 2009 11 R85 10.1186/ar2719 19500336 PMC2714136

[B62] Müller J Mollenhauer J Tuan R Benz K Quality control for mesenchymal stromal cells: chondrogenesis as a standard condition? Rheumatology: Current Research 2012 S3 003

