# Supplementary material for: Maintenance of “stem cell” features of cartilage cell sub-populations during in vitro propagation
Source: J Transl Med. 2013 Jan 30;11:27. doi: 10.1186/1479-5876-11-27 (PMC3637487; doi:10.1186/1479-5876-11-27)
Supplement: Additional file 1: Table S1 — Human tissues used for MACS / cell culture experiments. Table S2. Antibodies used for flow cytometry. Table S3. Primers used for gene expression analysis. Table S4. Gene expression of chondrocytic subpopulations dependent on the population doublings. Table S5. Adipogenic differentiation: relative gene expression data. Table S6. Osteogenic differentiation: relative gene expression data. Table S7. Chondrogenic differentiation: relative gene expression data. Table S8. Chondrogenic differentiation: GAG synthesis. [file 1479-5876-11-27-S1.doc]

**Supplementary tables**

Supplementary table 1: Human tissues used for MACS / cell culture experiments

| **No.** | **tissue** | **sex** | **age** | **medium primary culture** | **used in experiment** |
| --- | --- | --- | --- | --- | --- |
| Disc1 | Disc, vertebral replacement | female | 53 | CM, 10 % HS | Differentiation after expansion |
| Disc2 | Disc, vertebral replacement | male | 36 | CM, 10 % HS | Differentiation after expansion |
| Disc3 | Disc, vertebral replacement | female | 57 | CM, 10 % HS | Differentiation after expansion |
| Cho2 | OA cartilage | female | 53 | CM, 10 % FCS | Differentiation after expansion |
| Cho3 | OA cartilage | female | 55 | CM, 10 % FCS | Differentiation after expansion |
| Cho4 | OA cartilage | female | 58 | CM, 10 % FCS | Differentiation after expansion |
| Cho5 | OA cartilage | male | 75 | CM, 10% FCS | Differentiation after expansion |
| Cho6 | OA cartilage | female | 56 | CM, 10 % FCS | Chondrogenic state after sorting |
| Cho7 | Healthy donor cartilage | female | 67 | CM,10 % HS | Differentiation after expansion |
| Cho8 | Healthy donor cartilage | male | 75 | CM, 10 % HS | Differentiation after expansion |
| Cho9 | OA cartilage | female | 64 | CM, 10 % FCS | Chondrogenic state after sorting |
| Cho10 | OA cartilage | male | 54 | CM, 10 % FCS | Chondrogenic state after sorting |
| Cho11 | OA cartilage | female | 57 | CM, 10 % FCS | Only MACS |
| Cho12 | OA cartilage | female | 62 | CM, 10 % FCS | Flow cytometry after expansion (>8pds) |
| Cho13 | Healthy donor cartilage (cryo) | female | 61 | CM, 10 % HS | Differentiation of P0 cells after MACS ,  Flow cytometry after expansion( >8pds) |
| Cho14 | Healthy donor cartilage (cryo) | male | 69 | CM, 10 % HS | Differentiation of P0 cells after MACS,  Flow cytrometry after expansion (>8pds) |
| Cho15 | OA cartilage | female | 67 | CM, 10 % FCS | Differentiation of P0 cells after MACS |

Abbreviations:

CM chondrocyte medium

FCS fetal calf serum

HS human serum

OA osteoarthritis

cryo cryo-preserved cells (directly after isolation)

Supplementary table 2: Antibodies used for flow cytometry

| **Antibody** | **Recommended name** | **Purchaser** | **Dilution** |
| --- | --- | --- | --- |
| CD34 | Hematopoietic progenitor cell antigen | Milteny Biotech; Bergisch Gladbach, Germany | 1 : 11 |
| CD45 | Receptor-type tyrosine-protein phosphatase C, (Leukocyte common antigen) | Milteny Biotech; Bergisch Gladbach, Germany | 1 : 11 |
| CD49e | Integrin alpha-5 | BD Pharmigen; Heidelberg, Germany | 20 µl per test |
| CD73 | 5'-nucleotidase | Milteny Biotech; Bergisch Gladbach, Germany | 1 : 11 |
| CD90 | Thy-1 membrane glycoprotein | Milteny Biotech; Bergisch Gladbach, Germany | 1 : 11 |
| CD105 | Endoglin | Milteny Biotech; Bergisch Gladbach, Germany | 1 : 11 |
| CD140b | Platelet-derived growth factor receptor beta | BD Biosciences; Heidelberg, Germany | 20 µl per test |
| CD166 | Activated leukocyte cell adhesion molecule | BD Biosciences; Heidelberg, Germany | 20 µl per test |
| CD271 | Tumor necrosis factor receptor superfamily member 16 (Low-affinity nerve growth factor receptor) | Milteny Biotech; Bergisch Gladbach, Germany | 1 : 11 |
| W5C5 | Sushi domain-containing protein 2 (SUS2D) | Milteny Biotech; Bergisch Gladbach, Germany | 1 : 11 |
| W8B2 | Mesenchymal stem cell antigen (MSCA-1); tissue non-specific alkaline phosphatase (TNAP) | Milteny Biotech; Bergisch Gladbach, Germany | 1 : 11 |

Supplementary table 3: Primers used for gene expression analysis

| **Target gene** | **Accession number** | **Forward primer (5’ → 3’)** | **Reverse primer (5’ → 3’)** | **Amplicon (bp)** |
| --- | --- | --- | --- | --- |
| GAPDH | NM_002046.3 | AGAAAAACCTGCCAAATATGATGAC | TGGGTGTCGCTGTTGAAGTC | 126 |
| Collagen I (α2) | NM_000089.3 | GCTGGCAGCCAGTTTGAATATAAT | CAGGCGCATGAAGGCAAGT | 78 |
| Collagen II (α1) | NM_001844.3 | AGAGGTATAATGATAAGGATGTGTGGAAG | GTCGTCGCAGAGGACAGTCC | 83 |
| Aggrecan | NM_013227.2 | TGCATTCCACGAAGCTAACCTT | GACGCCTCGCCTTCTTGAA | 84 |
| Alk. Phosphatase | NM_000478.2 | TTCCCACGTCTTCACATTTGG | TTGCCATACAGGATGGCAGTG | 114 |
| Adiponectin | NM_004797.2 | CCATCTCCTCCTCACTTCCATT | AGTAGAACAGCTCCCAGCAACA | 93 |
| PPARy2 | NM_015869.4 | GGCCCTGGCAAAACATTTGTAT | CCCTCGCCTTTGCTTTGG | 68 |
| CBFA1 (RUNX2) | NM_001024630.3 | TGCCCGTGGCCTTCAAG | TGACAGTAACCACAGTCCCATCTG | 63 |
| SOX9 | NM_000346.3 | ATCCAAGCGCATTACCCACT | CGATTCTCCATCATCCTCCAC | 132 |

Supplementary table 4 (data in figure 6)

Table 4: Mean gene expression values relative to the expression of the reference gene GAPDH and standard deviations are presented. The unsorted and the W5C5 + / - fractions were pooled because no significant differences were obtained among these subgroups. Statistics: Logarithmical data were used. ANOVA was performed comparing the three groups at each phase of the culture separately. Significance p < 0.05.

Supplementary table 5 (data in figure 7) adipogenic differentiation: relative gene expression data

Significances indicated in figure 7. No significant differences were reached among subgroups (ANOVA).

Supplementary table 6 (data in figure 8) osteogenic differentiation: relative gene expression data

Significances indicated in figure 8. No significant differences were reached among subgroups (ANOVA).

Supplementary table 7 (data in figure 9) chondrogenic differentiation: relative gene expression data

Significances indicated in figure 9. No significant differences were reached among subgroups (ANOVA).

Supplementary table 8 (data in figure 9) chondrogenic differentiation: GAG synthesis
