# Supplementary material for: Maintenance of “stem cell” features of cartilage cell sub-populations during in vitro propagation
Source: J Transl Med. 2013 Jan 30;11:27. doi: 10.1186/1479-5876-11-27 (PMC3637487; doi:10.1186/1479-5876-11-27)
Supplement: Additional file 2: Figure S1 — Experimental set-up for MACS sorting experiments. Figure S2. Experimental set-up to characterise W5C5 and W8B2 positive subpopulations regarding MSC characteristics. [file 1479-5876-11-27-S2.doc]

**Supplemtentary figures**

**Figure 1:** **Experimental setup for MACS sorting experiments.**


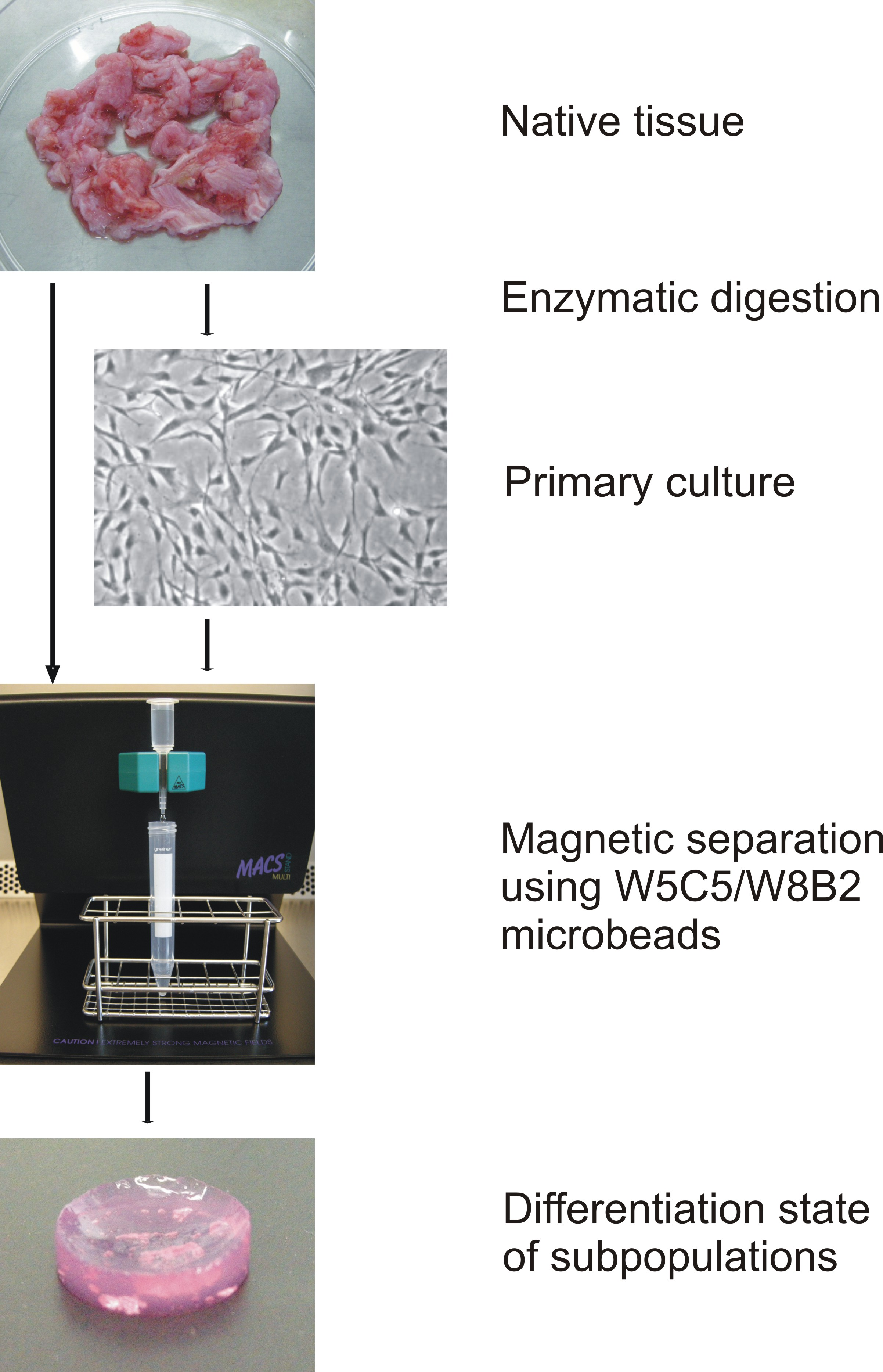


**Figure 2:** **Experimental set-up to characterise W5C5 and W8B2 positive subpopulations regarding MSC characteristics.**

**
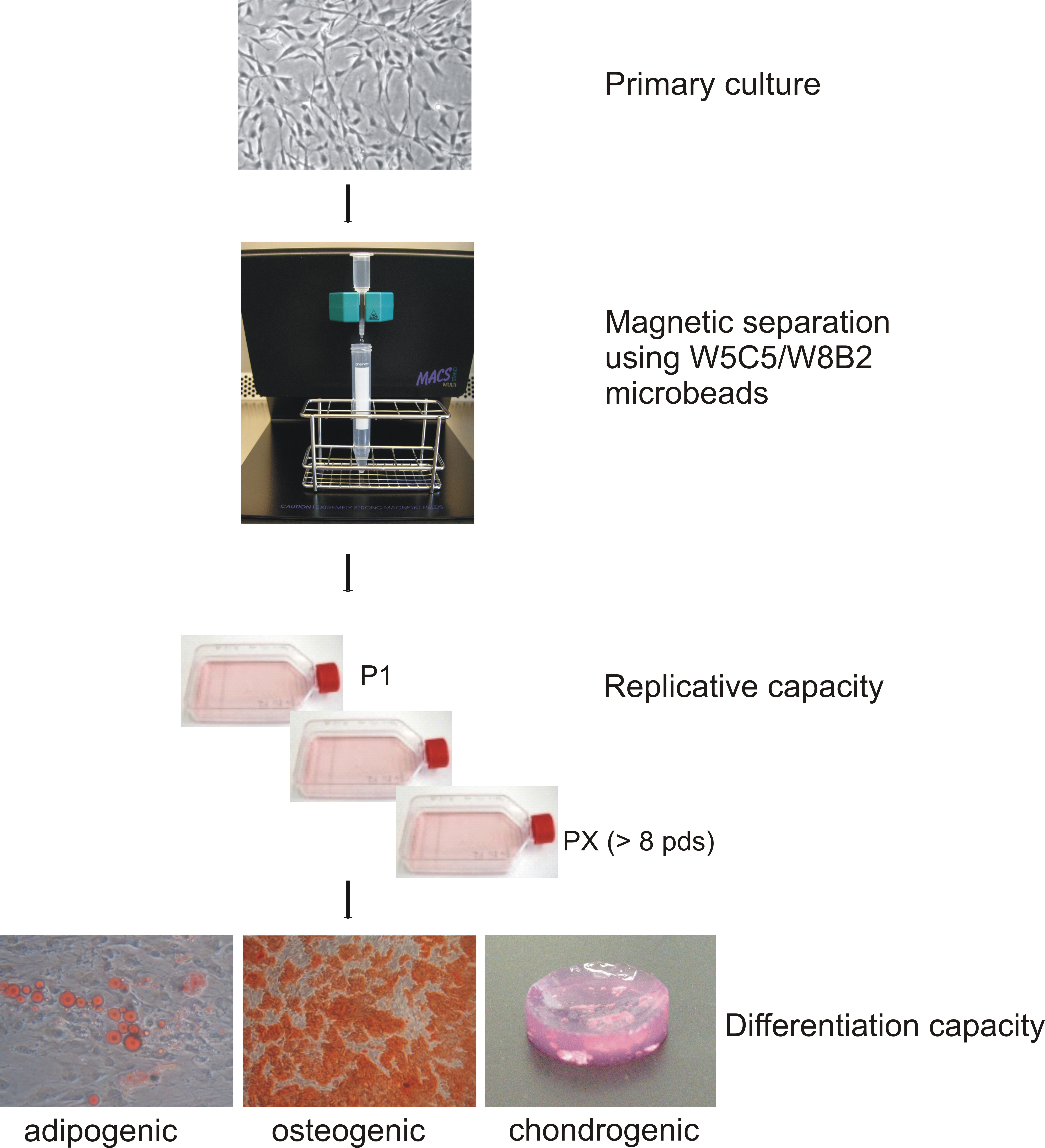
**
